# Vesicular Release of GABA by Mammalian Horizontal Cells Mediates Inhibitory Output to Photoreceptors

**DOI:** 10.3389/fncel.2020.600777

**Published:** 2020-12-01

**Authors:** Arlene A. Hirano, Helen E. Vuong, Helen L. Kornmann, Cataldo Schietroma, Salvatore L. Stella, Steven Barnes, Nicholas C. Brecha

**Affiliations:** ^1^Department of Neurobiology, David Geffen School of Medicine, University of California, Los Angeles, Los Angeles, CA, United States; ^2^Veterans Administration Greater Los Angeles Healthcare System, Los Angeles, CA, United States; ^3^Department of Ophthalmology, David Geffen School of Medicine, University of California, Los Angeles, Los Angeles, CA, United States; ^4^Doheny Eye Institute, University of California, Los Angeles, Los Angeles, CA, United States; ^5^Department of Medicine, David Geffen School of Medicine, University of California, Los Angeles, Los Angeles, CA, United States; ^6^Stein Eye Institute, David Geffen School of Medicine, University of California, Los Angeles, Los Angeles, CA, United States

**Keywords:** retina, horizontal cell, GABA, exocytosis, tonic current

## Abstract

Feedback inhibition by horizontal cells regulates rod and cone photoreceptor calcium channels that control their release of the neurotransmitter glutamate. This inhibition contributes to synaptic gain control and the formation of the center-surround antagonistic receptive fields passed on to all downstream neurons, which is important for contrast sensitivity and color opponency in vision. In contrast to the plasmalemmal GABA transporter found in non-mammalian horizontal cells, there is evidence that the mechanism by which mammalian horizontal cells inhibit photoreceptors involves the *vesicular release* of the inhibitory neurotransmitter GABA. Historically, inconsistent findings of GABA and its biosynthetic enzyme, L-glutamate decarboxylase (GAD) in horizontal cells, and the apparent lack of surround response block by GABAergic agents diminished support for GABA's role in feedback inhibition. However, the immunolocalization of the vesicular GABA transporter (VGAT) in the dendritic and axonal endings of horizontal cells that innervate photoreceptor terminals suggested GABA was released via vesicular exocytosis. To test the idea that GABA is released from vesicles, we localized GABA and GAD, multiple SNARE complex proteins, synaptic vesicle proteins, and Ca_v_ channels that mediate exocytosis to horizontal cell dendritic tips and axonal terminals. To address the perceived relative paucity of synaptic vesicles in horizontal cell endings, we used conical electron tomography on mouse and guinea pig retinas that revealed small, clear-core vesicles, along with a few clathrin-coated vesicles and endosomes in horizontal cell processes within photoreceptor terminals. Some small-diameter vesicles were adjacent to the plasma membrane and plasma membrane specializations. To assess vesicular release, a functional assay involving incubation of retinal slices in luminal VGAT-C antibodies demonstrated vesicles fused with the membrane in a depolarization- and calcium-dependent manner, and these labeled vesicles can fuse multiple times. Finally, targeted elimination of VGAT in horizontal cells resulted in a loss of tonic, autaptic GABA currents, and of inhibitory feedback modulation of the cone photoreceptor Ca_i_, consistent with the elimination of GABA release from horizontal cell endings. These results in mammalian retina identify the central role of vesicular release of GABA from horizontal cells in the feedback inhibition of photoreceptors.

## Introduction

Horizontal cells receive synaptic input from thousands of photoreceptors and feedback this broad spatial information back to photoreceptors as well as feeding it forward to bipolar cells to generate receptive field surrounds (Thoreson and Mangel, 2012). In 1970, Baylor et al. demonstrated that turtle retinal horizontal cells contribute a negative feedback signal to the cone photoreceptor light response. When our studies began over 30 years later, the proposed cellular mechanisms of horizontal cell neurotransmission were multiple, controversial, and unconventional: voltage- and sodium-dependent, calcium-independent plasmalemmal γ-aminobutyric acid (GABA) transporter (GAT) activity, as characterized in non-mammalian vertebrates (Schwartz, 2002), ephaptic coupling between photoreceptor calcium channel gating and current flow in horizontal cell glutamate receptors and hemichannels shown in fish retina (Byzov and Shura-Bura, 1986; Kamermans et al., 2001), and photoreceptor calcium current regulation by synaptic cleft pH (Hirasawa and Kaneko, 2003; Vessey et al., 2005; Cadetti and Thoreson, 2006; Kreitzer et al., 2012; Wang et al., 2014; Kramer and Davenport, 2015; Tchernookova et al., 2018; Grove et al., 2019). The apparent lack of the cone surround response block by GABAergic pharmacological agents in turtle, goldfish, mouse, and primate retinas (Thoreson and Burkhardt, 1990; Verweij et al., 1996, 2003; Endeman et al., 2012; Kemmler et al., 2014) was used to argue against a direct role for GABA in feedback inhibition. In contrast, early studies reported GABA in horizontal cells (Lam et al., 1978; Mosinger et al., 1986) suggesting that it may be a neurotransmitter used by horizontal cells. However, GABA immunoreactivity in horizontal cells was not consistently observed in adult mammalian retinas (Lam et al., 1978; Schnitzer and Rusoff, 1984; Mosinger et al., 1986; Chun and Wässle, 1989; Wässle and Chun, 1989) raising doubts about its role as a feedback transmitter. Further, unlike other GABAergic neurons, horizontal cells in adult mammalian retina did not take up ^3^H-GABA or ^3^H-muscimol (Blanks and Roffler-Tarlov, 1982; Wässle and Chun, 1989) and GATs were not expressed in these cells (Honda et al., 1995; Johnson et al., 1996; Casini et al., 2006; Guo et al., 2009, 2010). In contrast, horizontal cells in cat and monkey retinas showed GABA immunoreactivity (Agardh and Ehinger, 1982; Chun and Wässle, 1989; Pourcho and Owczarzak, 1989; Wässle and Chun, 1989; Grünert and Wässle, 1990). The GABA synthetic enzymes, glutamic acid decarboxylase65, and 67 (GAD65 and GAD67), were localized to mammalian horizontal cells (Vardi et al., 1994; Johnson and Vardi, 1998). Although like GABA, the GABA synthetic enzymes, GAD65 and GAD67 were observed in horizontal cells during development, and they were not consistently detected in adult horizontal cells (Brandon et al., 1979; Schnitzer and Rusoff, 1984; Mosinger and Yazulla, 1985). In contrast to the lack of GATs, the vesicular GABA transporter (VGAT/VIAAT, vesicular inhibitory amino acid transporter), which loads inhibitory amino acid transmitters into synaptic vesicles (McIntire et al., 1997; Sagné et al., 1997), was observed in amacrine and horizontal cells in multiple mammalian species (Haverkamp et al., 2000; Cueva et al., 2002; Jellali et al., 2002; Guo et al., 2010; Lee and Brecha, 2010; Hirano et al., 2011). The presence of VGAT in horizontal cell synaptic endings suggested that these unconventional neurons may release the neurotransmitter GABA via vesicular release. When VGAT was deleted from horizontal cells, these cells failed to feedback to photoreceptors (Hirano et al., 2016a) and the same mouse line revealed a lack of autaptic GABA reception by horizontal cells and no influence on cone calcium channels (Grove et al., 2019; Barnes et al., 2020), ending debate, at least in mammalian retinas, about whether horizontal cells utilize vesicular GABA release to send feedback to photoreceptors. Here we marshal evidence for the hypothesis that mammalian horizontal cells possess the cellular structures and proteins that mediate vesicular transmitter release. These include the presence and synthesis of GABA as a neurotransmitter, the essential molecular machinery for vesicular release, the structural basis of vesicular release, namely synaptic vesicles, and the regulated fusion and recycling of synaptic vesicles in mammalian horizontal cells. These findings show that the cellular mechanism underlying feedback inhibition in mammals involves vesicular GABA release by horizontal cells, and this stands to support a new GABA-pH hybrid model wherein autaptic reception of GABA by horizontal cells regulates pH in the synaptic cleft via depolarization and the bicarbonate permeability of the GABA receptors, resulting in the modulation of presynaptic calcium channels in photoreceptors (Grove et al., 2019; Barnes et al., 2020).

## GABA Is The Transmitter In Mammalian Horizontal Cells

### Presence of GABA in Horizontal Cells

**Figure 1:**
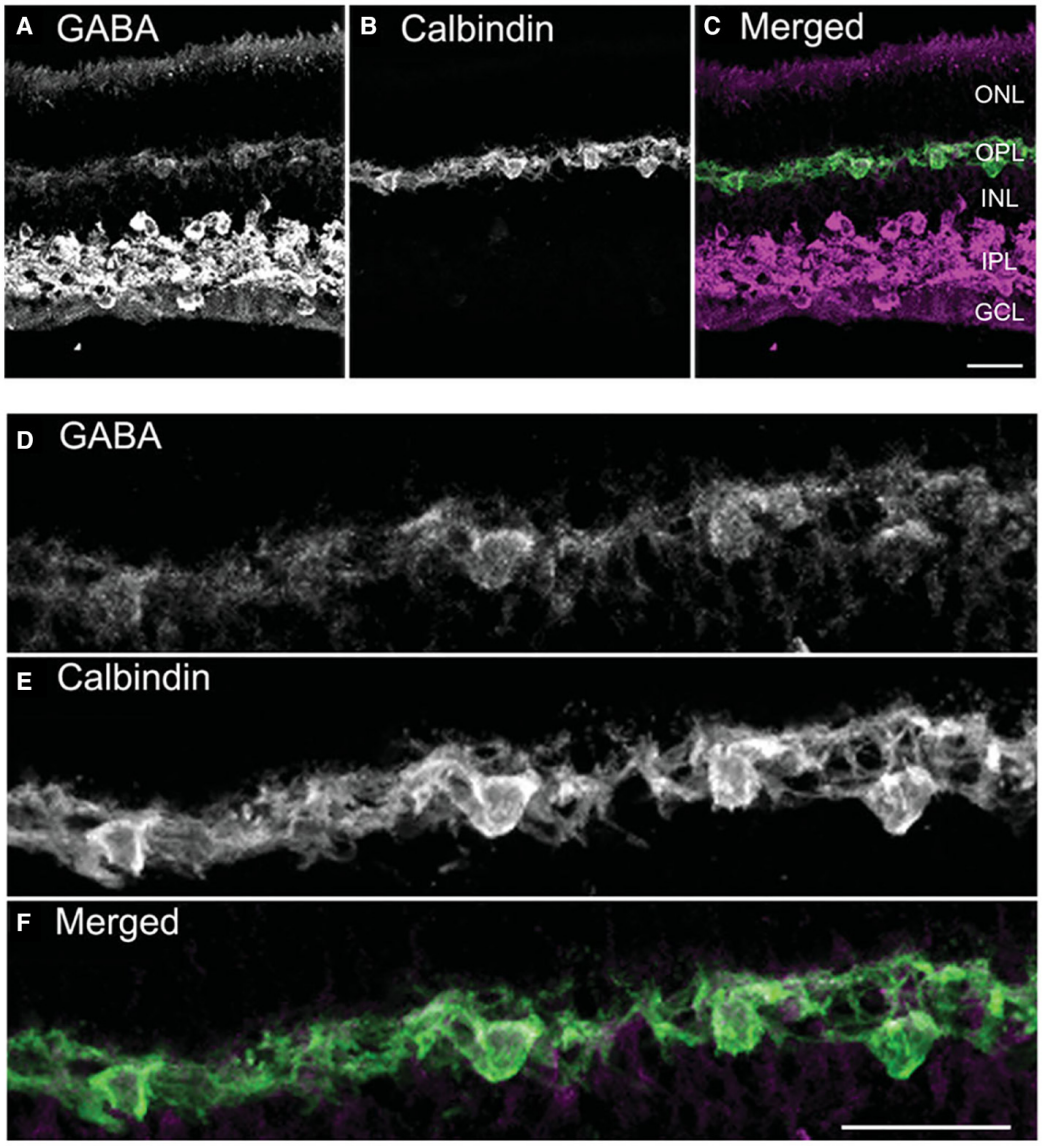
GABA immunoreactivity is localized to horizontal cell bodies and their processes. **(A–C)** A vertical section through the guinea pig retina was double labeled with antibodies to GABA **(A)** and calbindin-28K (calbindin, **B**). Weak, yet distinct, GABA immunolabeling occurs in the outer retina, in contrast to the strong GABA immunolabeling distributed to amacrine cell and displaced amacrine cell bodies and processes in the inner plexiform layer (IPL). **(C)** Merged image shows the co-localization in horizontal cell bodies and processes. **(D–F)** Higher magnification views of the outer plexiform layer (OPL) show the GABA immunoreactivity **(D)** in the calbindin-identified horizontal cells **(E)** in the merged image **(F)**. Images are maximum intensity projections of 6 optical sections, *z* = 5 μm. Scale bar, 20 μm in C (applies to **A–C**), **(F)** (applies to **D–F**). ONL, outer nuclear layer; INL, inner nuclear layer; GCL, ganglion cell layer. [Modified from (Guo et al., 2009)].

Several convergent findings show that GABA is the mammalian horizontal cell transmitter. Mammalian retinas contain typically two morphological types of horizontal cells, an axonless A-type whose dendrites contact only cones and an axon-bearing B-type whose dendrites contact cones and the axonal terminal system, the rods. Some rodents, including mouse and rat, possess only the B-type (Peichl and González-Soriano, 1994). The lack of immunoreactivity for GABA and its synthetic enzymes GAD65 and GAD67 in adult horizonal cells in some studies was used to argue against a role for GABA in horizontal cell neurotransmission. However, many studies have shown evidence for GABA in horizontal cells of cat, rabbit, rat, mouse, guinea pig, and primate retina (Nishimura et al., 1985; Mosinger et al., 1986; Osborne et al., 1986; Agardh et al., 1987; Mosinger and Yazulla, 1987; Wässle and Chun, 1988, 1989; Chun and Wässle, 1989; Pourcho and Owczarzak, 1989; Grünert and Wässle, 1990; Pow et al., 1994; Vardi and Auerbach, 1995; Kalloniatis et al., 1996; Johnson and Vardi, 1998; Koulen et al., 1998b; Guo et al., 2010; Deniz et al., 2011; Herrmann et al., 2011), albeit at lower levels than in amacrine cells (Pourcho and Owczarzak, 1989; Wässle and Chun, 1989; Vardi et al., 1994; Johnson and Vardi, 1998; Marc et al., 1998). In cat and monkey, horizontal cells in peripheral retina lacked GABA immunoreactivity, whereas they were immunoreactive in central retina (Wässle and Chun, 1989; Grünert and Wässle, 1990). Unlike non-mammalian horizontal cells in which not all subtypes contained GABA (Marc, 1992; Schwartz, 2002; Yang, 2004) both mammalian subtypes appeared to show GABA immunoreactivity (Wässle and Chun, 1989; Grünert and Wässle, 1990; Johnson and Vardi, 1998; Guo et al., 2010). In mouse and rabbit, horizontal cells exhibited high levels of GABA during early retinal development, which then dropped with maturation (Schnitzer and Rusoff, 1984; Osborne et al., 1986; Messersmith and Redburn, 1993; Pow et al., 1994). An example of the GABA immunolabeling is shown in horizontal cells of the adult guinea pig retina, which contains both A- and B-types (Figure 1, Guo et al., 2010) similar to cat and macaque retinas (Pourcho and Owczarzak, 1989; Wässle and Chun, 1989; Grünert and Wässle, 1990). GABA immunoreactivity, like the punctate staining of neurotransmitter receptors in retina (Wässle and Chun, 1989; Greferath et al., 1995) was highly sensitive to fixation conditions, favoring weak fixation (e.g., shorter fixation times, lower aldehyde concentrations) for visualization (Guo et al., 2010; Deniz et al., 2011). This lability as well as antibody specificity differences may account for reports of little to no immunostaining observed in well-fixed tissue (Agardh et al., 1986; Osborne et al., 1986; Versaux-Botteri et al., 1989; Messersmith and Redburn, 1992; Yamasaki et al., 1999; Loeliger and Rees, 2005).

### Glutamic Acid Decarboxylase (GAD)

**Figure 2:**
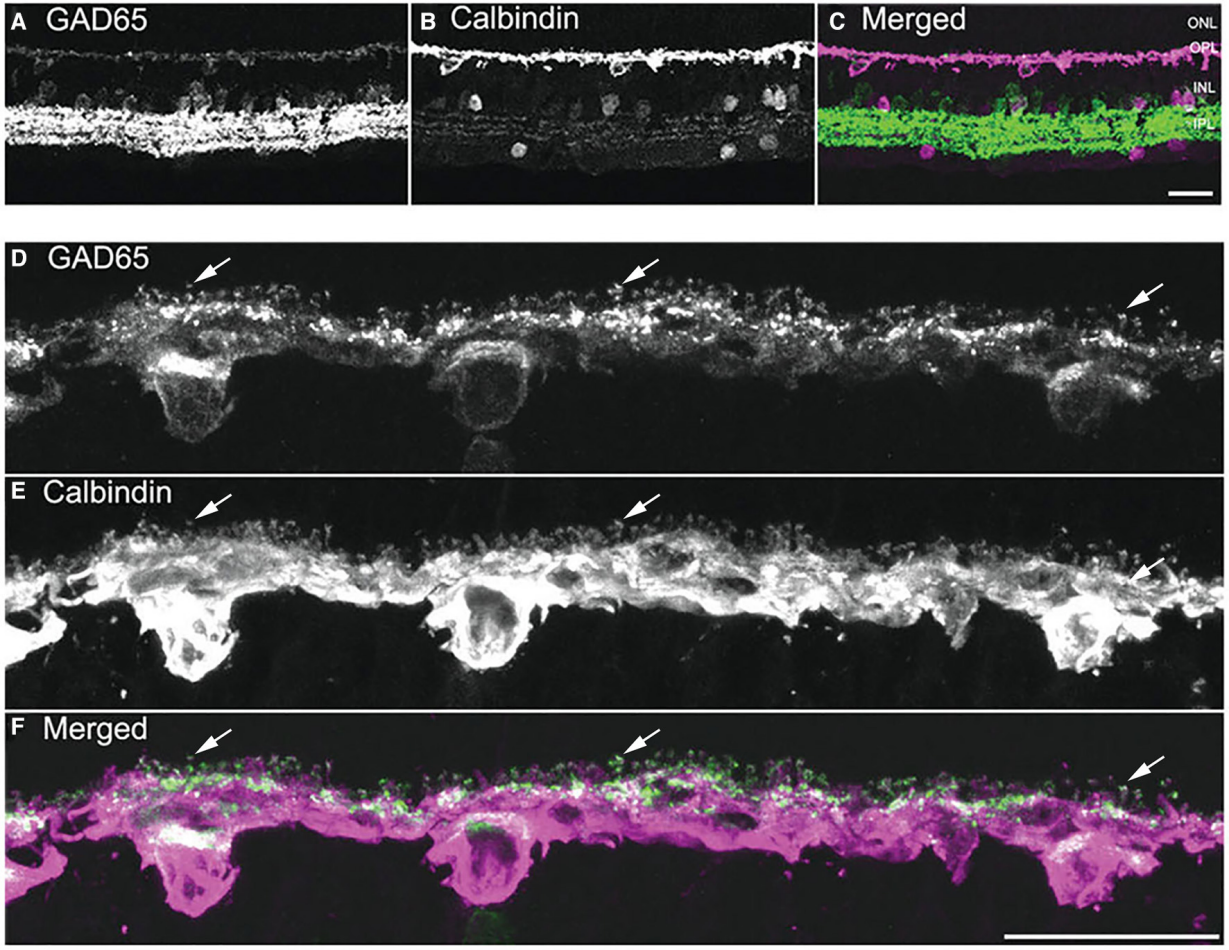
GAD65 immunoreactivity is localized to horizontal cells. **(A–C)** A vertical section through the guinea pig retina was double labeled with antibodies to GAD65 **(A)** and calbindin **(B)**. In the outer retina, weak GAD65 immunostaining is present in the cell bodies and processes in the OPL; whereas, strong GAD65 immunoreactivity is in amacrine cell and displaced amacrine cell bodies and processes in the IPL. **(B)** Horizontal cell somata and processes are labeled with calbindin antibodies. **(C)** Merged image demonstrates GAD65 immunostaining co-localized with calbindin immunostaining in the outer retina. **(D–F)** Higher magnification views of the OPL showing the localization of GAD65 immunoreactivity in horizontal cell somata, processes, and endings. **(D)** GAD65 immunolabeling in the OPL. **(E)** Calbindin immunolabeling of horizontal cells. **(F)** Merged image shows the co-expression of GAD65 and calbindin immunoreactivities in the OPL, indicating that GAD65 immunoreactivity is localized to horizontal cell bodies, processes, and tips. Arrows in **(D–F)** point to GAD65 in horizontal cell endings. Images are maximum intensity projections of 6 optical sections, *z* = 5 μm. Scale bar, 20 μm in **(C)** (applies to **A–C**), **(F)** (applies to **D–F**). [Modified from (Guo et al., 2009)].

The GABA-synthesizing enzyme L-glutamate decarboxylase (GAD) exists as two principal isoforms, GAD65 and GAD67 (Erlander et al., 1991; Kaufman et al., 1991). One or both of the GAD isoforms are found in mammalian horizontal cells at both the mRNA (Sarthy and Fu, 1989; Guo et al., 2010; Deniz et al., 2011) and protein levels (Schnitzer and Rusoff, 1984; Vardi et al., 1994; Vardi and Auerbach, 1995; Johnson and Vardi, 1998; Yamasaki et al., 1999; Dkhissi et al., 2001; Guo et al., 2010; Deniz et al., 2011). In rabbit retina, GAD65 and GAD67 immunoreactivities were detected in horizontal cells (Johnson and Vardi, 1998). Several studies report GAD67 immunostaining is present at high levels in horizontal cells of the developing and juvenile mouse, rat, and rabbit retina (Schnitzer and Rusoff, 1984; Osborne et al., 1986; Versaux-Botteri et al., 1989; Pow et al., 1994; Schubert et al., 2010), but at low or non-detectable levels in adult horizontal cells (Brandon et al., 1979; Schnitzer and Rusoff, 1984; Brandon, 1985; Osborne et al., 1986; Wässle and Chun, 1989; Brecha et al., 1991; Yazulla et al., 1997; Koulen et al., 1998b), including mouse (Haverkamp and Wässle, 2000; Schubert et al., 2010; Herrmann et al., 2011). GAD65 immunostaining (Figure 2) and mRNA were detected in adult guinea pig horizontal cells (Guo et al., 2010). Note the concentration of GAD65 immunoreactivity in the horizontal cell endings (Figure 2, arrows) and the scleral portion of the cell body. In rabbit horizontal cells, there are different subcellular localizations of GAD65 and GAD67 protein (Johnson and Vardi, 1998): GAD67 immunolabeling occurred in the dendritic terminals of A type and the dendritic and axonal terminals of the B type horizontal cells; whereas, GAD65 immunolabeling was found in A type somata and primary dendrites within the visual streak. In mouse, horizontal cells appear to express both GAD65 and GAD67 mRNA and protein (Deniz et al., 2011), but whether there is subcellular distribution difference between the two GAD isoform remains an open question.

The *Gad1* gene, encoding GAD67, is highly transcriptionally regulated by DNA methylation of the promoter, and exhibits alternative promoter usage and alternative splicing (Martin and Rimvall, 1993; Tao et al., 2018; Lee et al., 2019), that may account for some of the detection variability. Alternative splicing of *Gad1* produces proteins of differing molecular weights: the GAD67, GAD44, and GAD25 isoforms (Behar et al., 1993; Trifonov et al., 2014). Whereas GAD67 is thought to be constitutively active, GAD65 activity can be induced by neuronal activity (Lee et al., 2019). In the CNS, GAD65 is enriched in axonal terminals of GABAergic neurons (Esclapez et al., 1994). It is possible that the state of light adaptation and visual experience before collection of the tissue may influence the levels of protein detected (Connaughton et al., 2001). A transiently expressed GAD25/ES isoform was reported in retina (Connaughton et al., 2001; Dkhissi et al., 2001) and may account for the observed loss of GAD67 immunolabeling with retinal maturation. In addition to GAD67, there are at least 10 alternatively spliced isoforms of the full-length *Gad1* gene comprised of 19 exons, producing a GAD44 isoform that has enzymatic activity and several GAD25s that do not (Chessler and Lernmark, 2000; Liu et al., 2010; Trifonov et al., 2014; Tao et al., 2018). The *Gad2* gene encoding GAD65 appears to produce two splice variants, including a full-length mRNA and a truncated version of undefined function (Davis et al., 2016).

There is also post-transcriptional regulation of GAD, including palmitoylation, phosphorylation, and protein cleavage (Baekkeskov and Kanaani, 2009; Lee et al., 2019) that alters GAD protein activity and conformation, intracellular protein localization, and possibly antibody-targeted epitopes. GAD65 and GAD67 can form heterodimers, during targeting of GAD65 and GAD67 to synaptic vesicles in presynaptic terminals (Dirkx et al., 1995; Kanaani et al., 2010). GAD65 can form a complex with the synaptic vesicle proteins, VGAT, cysteine string protein, and heat shock protein 70 (Wei and Wu, 2008), and thus influence GABA loading into synaptic vesicles (Wei and Wu, 2008; Lee et al., 2019).

The detection of GAD or GABA in the adult retina may be influenced by numerous factors, including the differential expression of GAD isoforms, regulations of levels of *Gad* transcripts and GAD proteins, and GABA synthesis in horizontal cells, as well as technical issues related to fixation composition, fixation protocols (perfusion or immersion) and antibody specificity (Wässle and Chun, 1989; Pow and Crook, 1994; Vardi et al., 1994; Vardi and Auerbach, 1995; Kalloniatis et al., 1996; Johnson and Vardi, 1998; Deniz et al., 2011). Schubert et al. (2010) confirmed expression of GAD67 during neonatal development in mouse, but never detected GAD65 in horizontal cells. Some investigators observed the volatility of GABA immunoreactivity (Kalloniatis et al., 1996; Deniz et al., 2011) and suggested that it may be due to technical issues with harvesting the retina (Pow and Crook, 1994). GABA immunolabeling in mice was maintained by cardiac perfusion, but not post-dissection, fixation, and under physiological conditions that promoted GAD activity with L-glutamate/glutamine incubation with co-factor pyridoxal phosphate) or intracardiac perfusion with CNQX and cadmium to inhibit transmitter release from horizontal cells prior to fixation (Deniz et al., 2011).

There is evidence that GAD activity and/or level of expression may be regulated by light (Herrmann et al., 2011) and light adaptation (Connaughton et al., 2001) and this may contribute to inconsistencies in detection of GABA in horizontal cells. The GABA immunostaining in horizontal cells increased as mice were subjected to increasing intensity of background light (Herrmann et al., 2011), indicating light increased GABA immunoreactivity. In addition to changes in GAD activity, light stimulation of the retina would result in membrane hyperpolarization of horizontal cells and presumably less release of transmitter. In fish, the levels of the full-length GAD67 mRNA and protein (Connaughton et al., 2001) and GABA were increased in light-adapted retina (Lam, 1972; Starr, 1973; Connaughton et al., 2001). Finally, GAD65 and GAD67 mRNA expression in mouse horizontal cells is consistent with the GFP expression in GAD65-eGFP and GAD67-GFP adult reporter mice (Deniz et al., 2011). These findings suggest expression of both GAD65 and GAD67 in adult mouse horizontal cells occurs (Deniz et al., 2011), but see (Schubert et al., 2010).

### GABA Receptors in Outer Retina

The localization of GABA receptors in the outer retina to photoreceptors, bipolar cells, and horizontal cells (Brecha, 1992; Yang, 2004) is congruent with both feedback and feed-forward roles for GABA released from horizontal cells. In non-mammalian retina, such as turtle, fish and salamander, photoreceptors clearly possess functional GABA_A_ receptors, as GABA application generated a chloride conductance (Wu and Dowling, 1980; Tachibana and Kaneko, 1984; Kaneko and Tachibana, 1986; Yazulla et al., 1989; Wu, 1992). Reports of clear-cut expression of GABA_A_ receptors in mammalian photoreceptors are scant, although there are reports of GABA_A_ receptor subunit mRNAs by photoreceptors by *in situ* hybridization (ISH), single-cell RT-PCR, and GABA_A_ receptor subunit immunohistochemistry (Greferath et al., 1993; Grigorenko and Yeh, 1994; Vardi et al., 1998). In rat retina, GABA_A_ receptor subunit α2 is reported to be expressed at cone photoreceptor terminals and the β1, δ, γ2 mRNAs are expressed in the outer nuclear layer (ONL) (Greferath et al., 1995). However, the α1 subunit mRNA was not detected in the ONL of rat retina (Brecha et al., 1991), consistent with the lack of α1 and ρ1 immunoreactivities in mouse cone pedicles by immunoelectron microscopy (Kemmler et al., 2014). In neonatal rabbit retina, cone photoreceptors transiently express GABA_A_ receptor subunits α1 and β2/3 (Mitchell and Redburn, 1996; Mitchell et al., 1999), when GABA and GAD67 levels are high in horizontal cells (Schnitzer and Rusoff, 1984). Cone terminals of pig and rat were reported to show GABA_A_ρ subunit (ρ subunit) immunoreactivity suggesting the presence of a GABA_A_ρ receptor (Picaud et al., 1998b; Pattnaik et al., 2000). However, Deniz et al. (2019) reported bicuculine-sensitive, but not TPMPA-sensitive, GABA evoked currents in mouse cone photoreceptors in retinal slices, suggesting the presence of ionotropic GABA_A_ receptors, but not those comprising ρ-subunits. Rod photoreceptors from cultured pig retina and in mouse retinal slices were reported to exhibit no response to GABA (Picaud et al., 1998b; Deniz et al., 2019).

**Figure 3:**
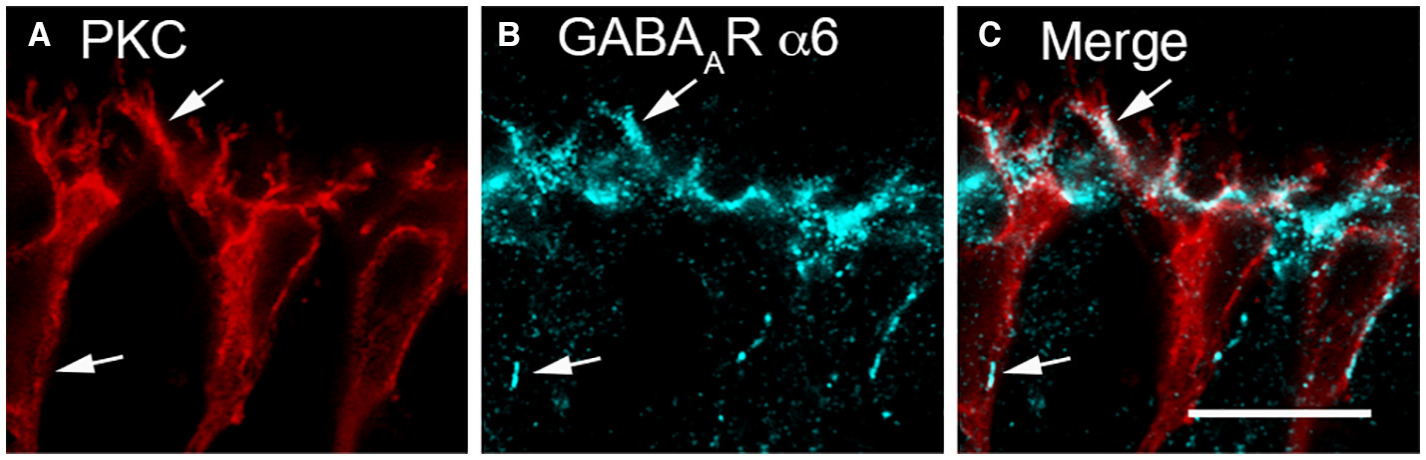
GABA_A_R α6 subunit immunolabeling occurred in bipolar cell dendrites. **(A–C)** Rod bipolar cells bear α6 immunoreactivity on their dendrites. **(A)** PKCα antibodies identify rod bipolar cells (red). **(B)** α6 immunolabeling (blue) occurs in patches along the bipolar cell dendrites (arrows) in the OPL and in the cell body membrane (arrows). **(C)** Merged image demonstrates co-localization of α6 and PKCα immunoreactivities in the dendrites of rod bipolar cells (arrows) and to a lesser degree on their somata (arrows). Single optical section **(A–C)**. Scale bar, 10 μm. (Modified from Hirano et al., 2016b).

Evidence for a horizontal cell feed-forward role includes the expression of GABA_A_ receptor immunoreactivity on bipolar cell dendrites (Wässle and Chun, 1989; Vardi et al., 1992; Greferath et al., 1993; Brecha and Weigmann, 1994; Vardi and Sterling, 1994; Enz et al., 1996; Wässle et al., 1998; Haverkamp and Wässle, 2000; Haverkamp et al., 2000; Hoon et al., 2015). GABA_A_ receptor subunit immunoreactivity is localized to bipolar cell membranes adjacent to horizontal cell endings in cone pedicles and underneath the photoreceptor terminals (Greferath et al., 1994; Vardi and Sterling, 1994; Koulen et al., 1998a; Haverkamp et al., 2000; Puller et al., 2014). The extrasynaptic GABA_A_ receptor α6 subunit is expressed on rod bipolar cell dendrites (Figures 3A–C) (Hirano et al., 2016b), which suggests a role for tonic GABA_A_ receptor currents in feedforward signaling.

As functional evidence of a feedforward input, full-field light stimulation, applied in the presence of L-AP4 to block direct photoreceptor input, reduced a gabazine-sensitive current in ON-cone bipolar cells (Yang and Wu, 1991; Chaffiol et al., 2017). This feedforward input results from GABA_A_ receptor activation at ON cone bipolar cell dendrites, which is reduced by horizontal cell hyperpolarization. GABA may evoke responses of opposite polarities in ON and OFF bipolar cells as a result of differing internal chloride concentrations in their dendrites (Duebel et al., 2006). GABA elicited depolarizing inward currents when applied to dendrites of mouse rod bipolar cells and hyperpolarizing currents when applied to OFF-bipolar cells, congruent with feedforward input from horizontal cells (Satoh et al., 2001; Duebel et al., 2006). The basis of the differential intracellular chloride is the expression of Na^+^-K^+^-Cl^−^ co-transporter (NKCC), which transports chloride into the cellular compartment, which is prominent in ON bipolar cell dendrites and horizontal cells (Vardi et al., 2000; Dmitriev et al., 2007; Puller et al., 2014). NKCC promotes accumulation of intracellular chloride and generates a chloride equilibrium potential above the resting membrane potential and thus a depolarization when ionotropic GABA receptor chloride channels are opened. In contrast, K^+^-Cl^−^ co-transporter (KCC2), a chloride extruder, is expressed in OFF bipolar cell dendrites and axonal terminals of ON and OFF bipolar cells (Vardi et al., 2000), where a GABA-activated chloride conductance would elicit a hyperpolarization.

Finally, GABA released by horizontal cells appears to act back on the horizontal cells themselves (Kamermans and Werblin, 1992; Blanco et al., 1996; Feigenspan and Weiler, 2004; Varela et al., 2005; Thoreson and Mangel, 2012). In non-mammalian horizontal cells, GABA elicited currents by activating ionotropic GABA_A_ receptors, including GABA_Aρ_ receptors, or electrogenic transporters [fish: (Wu and Dowling, 1980; Schwartz, 1982; Gilbertson et al., 1991; Kamermans and Werblin, 1992; Cammack and Schwartz, 1993; Qian and Dowling, 1993; Takahashi et al., 1994, 1995; Jung et al., 1999) salamander: (Yang and Wu, 1993; Dong and Werblin, 1994; Yang et al., 1999; Wang et al., 2000)]. GABA elicited ionotropic GABA_A_ receptor-mediated currents in mammalian (rabbit, mouse, rat, human) horizontal cells, but not a transporter-mediated current (Blanco et al., 1996; Picaud et al., 1998a; Feigenspan and Weiler, 2004; Varela et al., 2005; Liu et al., 2013). GABA and/or muscimol application activated ionotropic GABA_A_ receptors and elicited chloride currents, blocked by bicuculline and picrotoxin, in whole-cell recordings of isolated rabbit, mouse, and rat horizontal cells (Blanco and de la Villa, 1999; Feigenspan and Weiler, 2004; Liu et al., 2013). In mouse horizontal cells, we showed distinct immunolabeling for GABA_A_ρ ρ2 subunit localized predominantly to their endings at its axon terminals within rod spherules and at its dendrites at cone pedicles (Grove et al., 2019; Barnes et al., 2020), indicating the presence of GABA_A_ρ receptors. Notable characteristics of GABA_A_ρ receptors include high affinity for GABA and non-desensitizing currents, capable of producing tonic currents at ambient levels of interstitial GABA, similar to extrasynaptic GABA receptors in other areas of the CNS (Bormann and Feigenspan, 1995; Bormann, 2000; Farrant and Nusser, 2005). Horizontal cells, recorded in rodent (mouse, rat, guinea pig) retinal slices, maintained a tonic GABA current in the cone terminal synaptic cleft that was sensitive to TPMPA, a GABA _A_ρ receptor blocker, and this tonic current proved critical for feedback inhibition of cone calcium current (Grove et al., 2019). Recordings in a horizontal cell conditional knockout of VGAT showed this tonic GABA current was abolished in these horizontal cells (Grove et al., 2019), suggesting that horizontal cells were the source of the GABA. In addition to ionotropic GABA receptors, metabotropic GABA_B_ receptors have been reported on rat horizontal cell processes (Koulen et al., 1998b). Taken together, these studies indicate multiple targets for GABA exist in the OPL, which could mediate the action of horizontal cells in the outer retina.

### GABA Transporter (GAT)

Earlier models of GABA release from non-mammalian horizontal cells posited a central role for a Ca-independent, Na-dependent GABA transporter, GAT-1 (Schwartz, 1987; 2002). GABA uptake or release from the cytoplasm (Schwartz, 2002) is unlikely in mammalian horizontal cells based on several findings. First, uptake studies using radiolabeled GABA or GABA analogs have not reported high affinity uptake of these molecules by adult horizontal cells, although high affinity uptake was readily observed in amacrine cells (Ehinger, 1977; Agardh and Ehinger, 1982; Blanks and Roffler-Tarlov, 1982; Mosinger et al., 1986; Pow et al., 1996). In addition, GABA transporter currents have not been detected in isolated mouse and rabbit horizontal cells (Feigenspan and Weiler, 2004; Varela et al., 2005). These findings are consistent with the failure to detect GAT mRNAs and immunostaining in horizontal cells of mouse, rat, and guinea pig retinas (Brecha and Weigmann, 1994; Honda et al., 1995; Johnson et al., 1996; Guo et al., 2009). In mammalian retinas, GAT-1 and GAT-3 instead are expressed by Müller cells (Johnson et al., 1996; Guo et al., 2009) that take up [^3^H]-GABA (Marshall and Voaden, 1975; Blanks and Roffler-Tarlov, 1982).

In mammals, a preponderance of evidence shows that GABA meets the criteria for being a neurotransmitter of horizontal cells. There is the synthetic machinery for GABA in horizontal cells, detectable GABA immunoreactivity, and a plethora of GABA receptors in the OPL that would mediate the action of the released GABA (Wässle et al., 1998; Haverkamp et al., 2000). While mammalian horizontal cells do not express GATs, GABA uptake occurs in Müller cell processes that surround photoreceptor terminals, producing a honeycomb pattern in the outer plexiform layer (OPL) (Burris et al., 2002; Guo et al., 2009).

## Presence of Proteins Involved In Vesicular Release

### Vesicular Neurotransmitter Transporter

**Figure 4:**
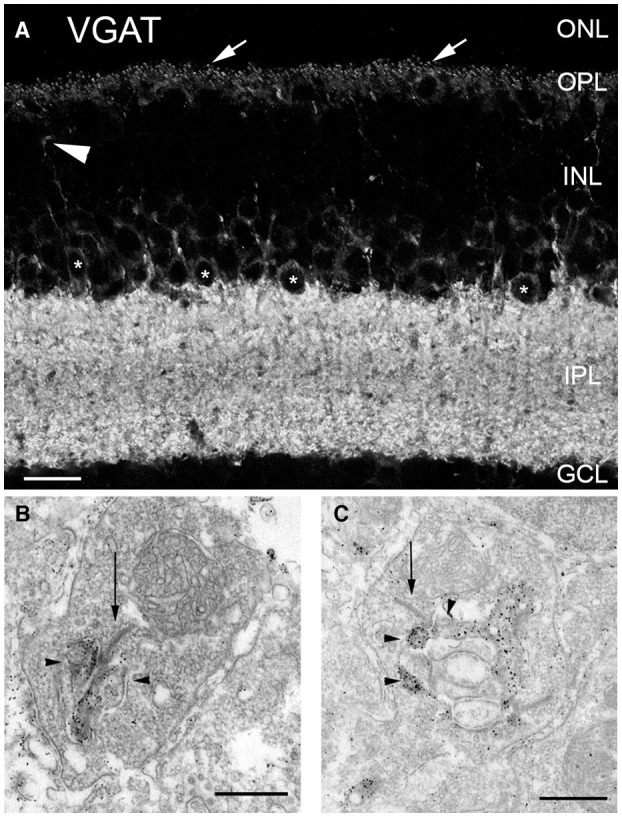
Vesicular γ-aminobutyric acid transporter (VGAT) was present in horizontal cell endings. **(A)** VGAT antibody staining of a vertical section of mouse retina showed labeled puncta (arrows), weak immunolabeling in the OPL and strong immunolabeling in the IPL, and around cell bodies (*) of the proximal inner nuclear layer (INL). Arrowhead points to a VGAT-containing interplexiform process. ONL, outer nuclear layer; GCL, ganglion cell layer. Scale bar, 20 μm. **(B,C)** VGAT immunoreactivity is localized in horizontal cell synaptic endings at photoreceptor synapses. Electron micrographs illustrate the dark and granular DAB reaction product of the VGAT immunoreactivity in terminals of horizontal cells of mouse **(B)** and rat **(C)** retina. Arrows indicate photoreceptor synaptic ribbons. Arrowheads indicate horizontal cells. Scale bars, 0.5 μm in **(B,C)**. (Modified from (Cueva et al., 2002).

VGAT is a transporter that accumulates inhibitory amino acid transmitters into synaptic vesicles in GABA- and glycine-containing neurons (McIntire et al., 1997; Sagné et al., 1997; Chaudhry et al., 1998; Gasnier, 2000). Whereas mammalian horizontal cells lack plasmalemmal GATs (Johnson et al., 1996; Guo et al., 2009), our laboratory and others showed the presence of the vesicular inhibitory amino acid/GABA transporter (VIAAT/VGAT) in mammalian horizontal cells in mouse (Figure 4A), rat, rabbit and primate retina, where VGAT immunostaining is concentrated in the endings that insert into the rod and cone photoreceptor terminals (Figure 4A, arrows, Haverkamp et al., 2000; Cueva et al., 2002; Jellali et al., 2002; Johnson et al., 2003; Hirano et al., 2005, 2007, 2011; Guo et al., 2010; Lee and Brecha, 2010). Note also the labeled interplexiform process of a tyrosine hydroxylase (TH) amacrine cell (Figure 4A, arrowhead, Witkovsky et al., 2008) and strongly immunolabeled interplexiform layer (IPL) and amacrine cell somata (^*^). Ultrastructural analysis showed that VGAT immunolabeling was found in the horizontal processes that form the lateral elements at mouse and rat photoreceptor synapses (Figures 4B,C, Cueva et al., 2002). This VGAT localization to synaptic endings suggested that mammalian horizontal cells released GABA via vesicular exocytosis for signaling.

### SNARE Proteins

**Figure 5:**
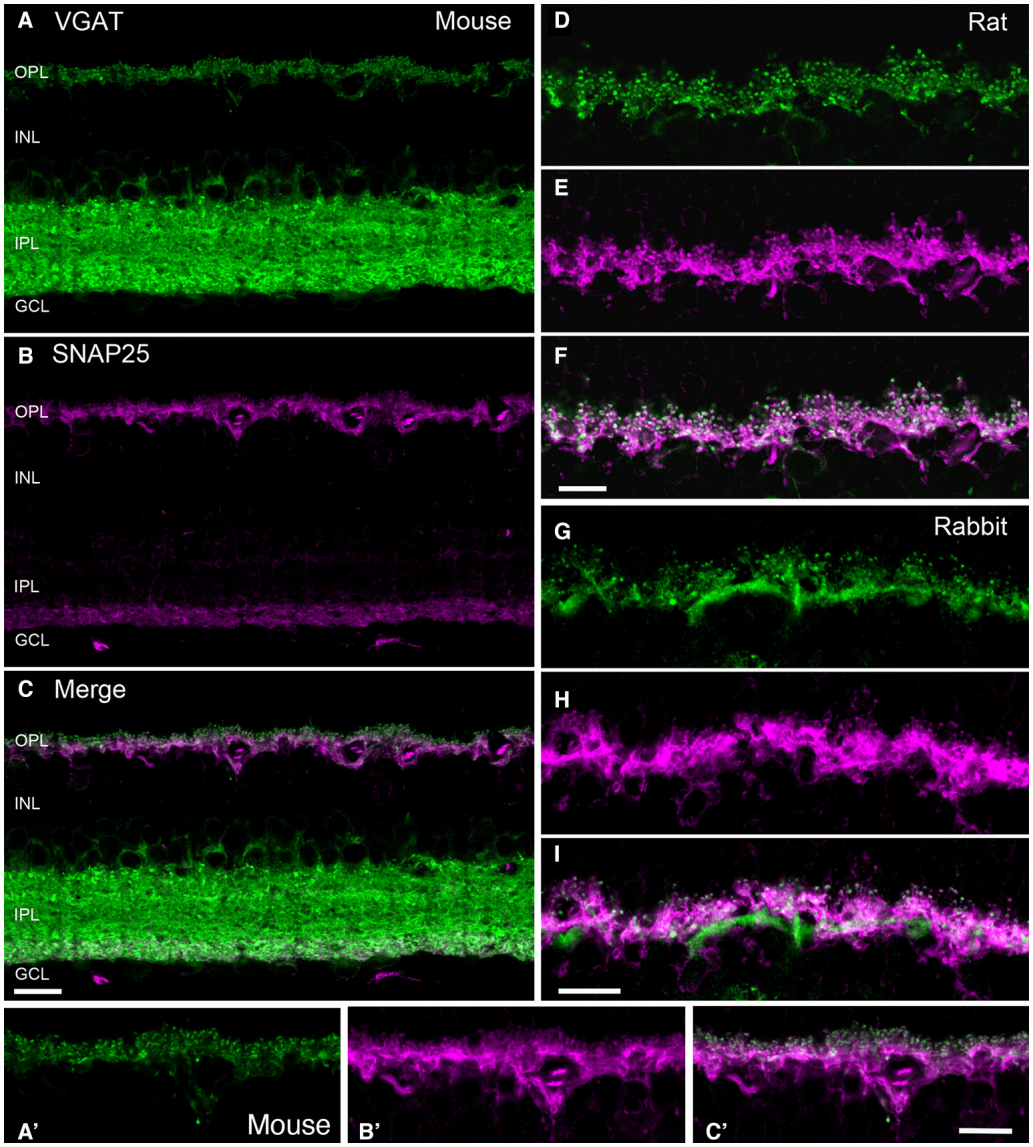
SNARE protein SNAP-25 co-localized with VGAT in horizontal cell processes in mammalian retina. VGAT antibody staining (green) of a vertical section of mouse **(A,A')**, rat **(D)**, and rabbit **(G)** retinas showed immunolabeling in the OPL and the IPL, and around cell bodies of the proximal inner nuclear layer (INL). SNAP-25 antibody labeling (magenta) of the same section produced immunolabeling in the OPL and the proximal IPL of mouse **(B)**, and OPL of mouse **(A')**, rat **(E)**, and rabbit **(H)** retinae. Merged images of the VGAT and SNAP-25 immunolabeling (white) indicated co-localization of SNAP-25 with VGAT in the tips of horizontal cells in mouse **(C,C')**, rat **(F)**, and rabbit **(I)**. GCL, ganglion cell layer. Maximum intensity projections. Scale bar, 10 μm in **(C)** (applies to **A–C**), **(F)** (applies to **D–F)**, and **(I)** (applies to **G–I**). (Modified from Hirano et al., 2011).

The core complex for fusion of synaptic vesicles with the plasma membrane consist of three soluble N-ethylmaleimide-sensitive factor attachment protein (SNAP) receptor (SNARE) proteins: two are plasma membrane proteins, syntaxin-1 and SNAP-25, and the third is the vesicle-associated membrane protein (VAMP-2)/synaptobrevin-2 (Jahn and Scheller, 2006; Südhof, 2013; Yoon and Munson, 2018). Horizontal cell endings contain the SNARE protein isoforms: SNAP-25 (Hirano et al., 2011), syntaxin-1a (Hirano et al., 2005), and VAMP-1, that likely interact to form the minimal machinery for membrane fusion. Figure 5 depicts double labeling for VGAT and SNAP-25, which shows co-localization in horizontal cells in the OPL of mouse (Figures 5A-C,A′-C′), rat (Figures 5D-F), and rabbit retina (Figures 5G-I).

**Figure 6:**
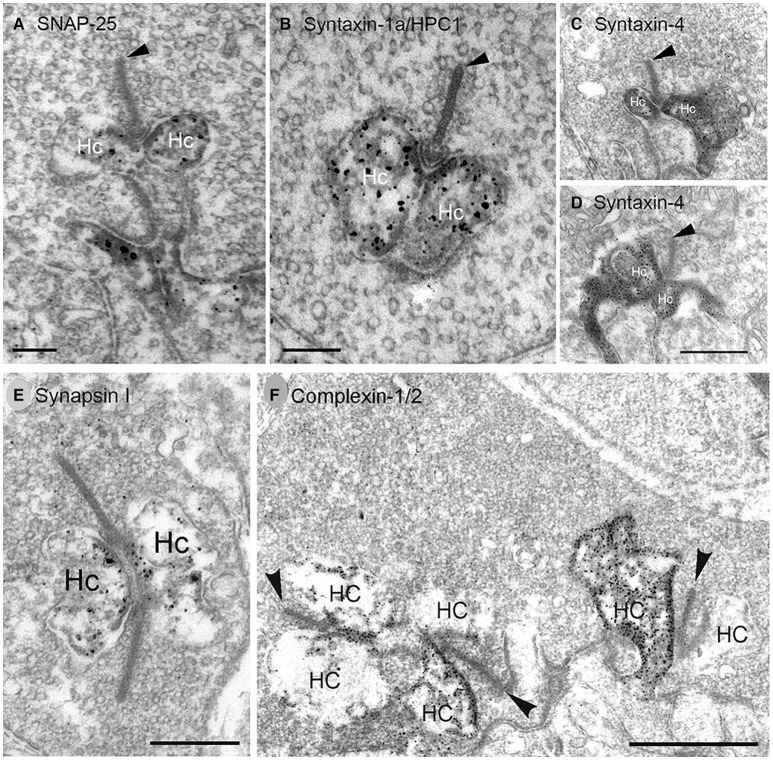
SNARE complex and synaptic proteins localize to horizontal cell synaptic endings. Pre-embedding immunoelectron microscopy with antibodies to **(A)** SNAP-25, **(B)** syntaxin-1a/HPC1, **(C,D)** syntaxin-4, **(E)** synapsin I, **(F)** complexin-1/2 produced dark, granular DAB immunolabeling for each SNARE (**A–D**, SNAP-25, syntaxin-1a, syntaxin-4) or synaptic protein (**E,F**, synapsin I, complexin-1/2) in horizontal cell (Hc) endings at **(A–E)** rod photoreceptor synapses. **(F)** Complexin-1/2 labeling of lateral elements at cone photoreceptor synapses. Arrowheads point to synaptic ribbons. **(A,B,E,F)**, rabbit retina; **(C,D)**, mouse retina. Scale bars, 0.2 μm in **(A)**, 0.3 μm in **(B)**, 0.5 μm in **(C,D)**, 0.4 μm in **(E)**, 1 μm in **(F)**. (Modified from **(A)** Hirano et al., 2011; **(B,E,F)** Hirano et al., 2005; **(C,D)** Hirano et al., 2007).

While there were consistent reports of SNAP-25 immunoreactivity in the IPL of mammalian retinas (Catsicas et al., 1992; Ullrich and Südhof, 1994; Brandstätter et al., 1996b; Grabs et al., 1996; Von Kriegstein et al., 1999; Greenlee et al., 2001), there were contradictory reports of its cellular distribution in the outer retina. SNAP-25 immunostaining was reported in horizontal cells of several mammalian species (mouse, rat, monkey, cow) (Catsicas et al., 1992; Grabs et al., 1996; Von Kriegstein et al., 1999; Greenlee et al., 2001). In contrast, other studies reported SNAP-25 immunoreactivity in rat photoreceptor terminals, but not horizontal cells (Ullrich and Südhof, 1994; Brandstätter et al., 1996b; Morgans et al., 1996). Our studies (Lee and Brecha, 2010; Hirano et al., 2011) showed consistent SNAP-25 immunostaining in mouse (Figure 5B), rat (**E**) and rabbit (**H**) horizontal cells, identified by calbindin immunoreactivity (Röhrenbeck et al., 1987), with multiple SNAP-25 antibodies (Hirano et al., 2011). SNAP-25 co-localized with VGAT in all three species (Figure 5) and SNAP-25 immunolabeling was found ultrastructurally in horizontal cell processes at photoreceptor terminals (Figure 6A, Hirano et al., 2011). Not surprisingly, as SNAP-25 participates in vesicle trafficking in multiple cellular pathways, one of the SNAP-25 antibody (SMI-31) labeled all retinal cell types (Hirano et al., 2011). Differences in retinal SNAP-25 labeling patterns may be due to the expression of two isoforms of SNAP-25a and b, one of which confers a palmitoylation site for plasma membrane anchoring (Hirano et al., 2011).

**Figure 7:**
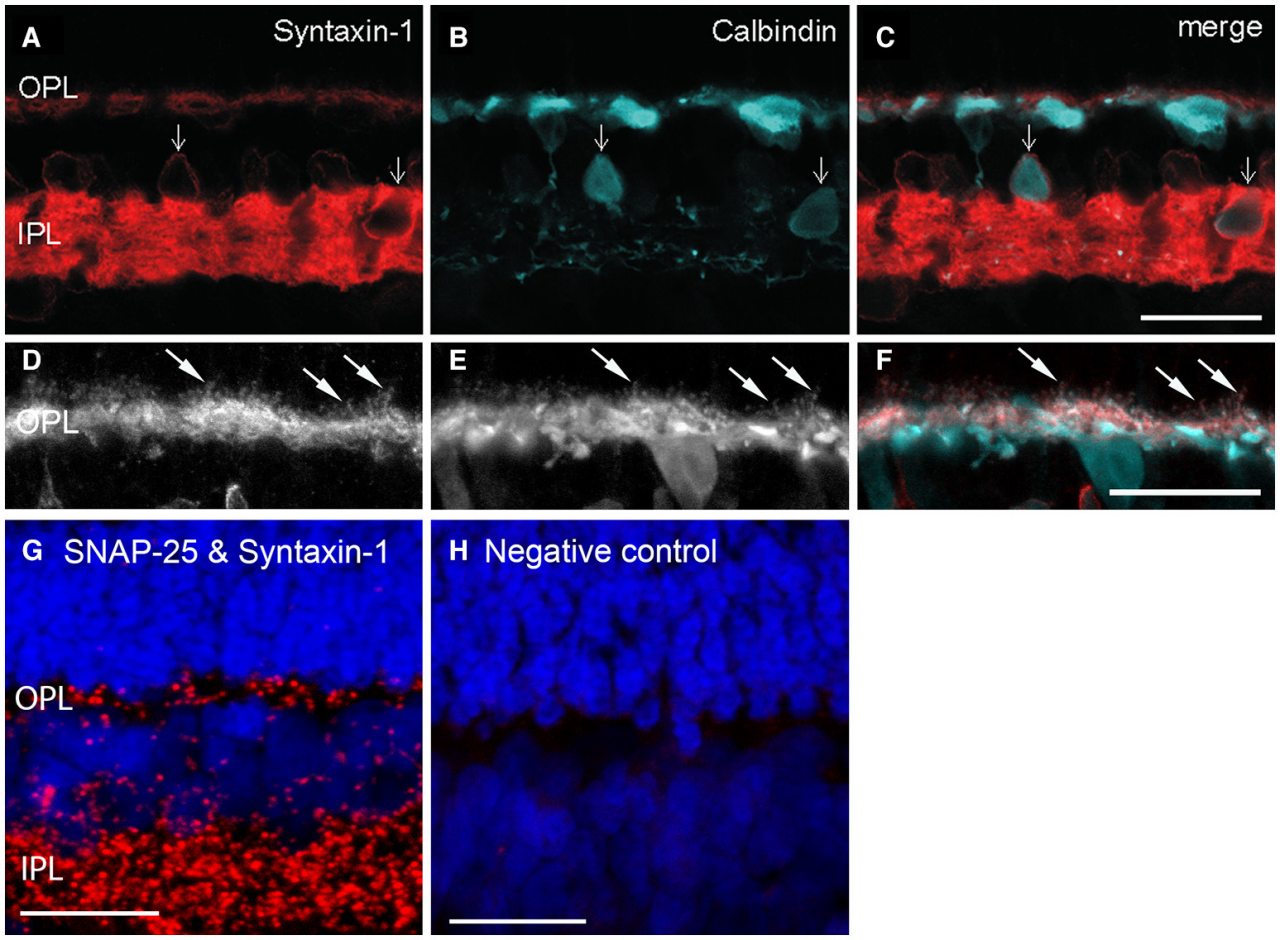
Cellular localization of syntaxin-1a to horizontal cells and their processes and endings in photoreceptor synapses. **(A)** Syntaxin-1a (red) immunolabeling co-localized with that of **(B)** calbindin (blue) in horizontal cell bodies and processes in the OPL **(C)**, as well as some amacrine cell bodies (arrows) in the INL in rabbit retina. In addition to horizontal cells, calbindin immunoreactivity was present in a subtype of bipolar cell and amacrine cells in rabbit retina. OPL, outer plexiform layer; IPL, inner plexiform layer. **(D–F)** Higher magnification views of immunolabeling for syntaxin-1a **(D)**, calbindin **(E)**, and merged image **(F)** in the OPL of rabbit retina. Arrows point to horizontal cell endings. **(A–C)** Single optical section; **(D–F)** maximum intensity projection, *z* = 2.88 μm. (Modified from Hirano et al., 2005). **(G,H)** *In situ* proximity ligation assay (PLA) revealed protein interactions between the plasma membrane SNARE proteins SNAP-25 and syntaxin-1a in both plexiform layers. **(G)** *In situ* PLA marks close (within 40 nm) protein interactions and identifies these interactions as distinct puncta that are localized to the OPL and more densely in the IPL of guinea pig retina. **(H)** Negative controls in which one of the antibodies was omitted resulted in no puncta. **(G,H)** Confocal images were scanned at 0.5 μm intervals, and maximum intensity projection of 9 optical images, *z* = 4.0 μm. Scale bar, 20 μm (**A–C** in **C**, **D–F** in **F**, **G,H**). (Modified from Brecha et al., 2010).

Syntaxin-1 to−4 direct vesicle targeting to the plasma membrane, with syntaxin-1 typically specialized for presynaptic membranes (Teng et al., 2001; Südhof, 2004; Sherry et al., 2006; Rizo and Xu, 2015). Syntaxin-1a is highly expressed in amacrine cells (Barnstable et al., 1985), but Syntaxin-1a/HPC1 is also present in horizontal cells, albeit at lower levels (Figures 7A–F, Brandstätter et al., 1996b; Morgans et al., 1996; Greenlee et al., 2001; Hirano et al., 2005; Lee and Brecha, 2010), as well as in interplexiform cell processes in the OPL (Brandstätter et al., 1996b; Morgans et al., 1996). Our pre-embedding immunoelectron microscopy findings show syntaxin-1a immunoreactivity in mammalian horizontal cell endings (Figure 6B). *In situ* proximity ligation assay (PLA, Biolink Bioscience) employs specific antibodies against potentially interacting proteins and visualizes these close (within 40 nm) interactions with oligonucleotide-conjugated secondary antibodies to identify individual sites of protein-protein interactions at the cellular level in tissue sections (Söderberg et al., 2006, 2008). Using PLA, we have shown that SNAP25 and syntaxin-1a are located within 40 nm of each other (red puncta) in the OPL, indicating that they are likely binding partners in horizontal cell processes (Figures 7G,H, Brecha et al., 2010). This interaction likely occurs in the horizontal cells in the OPL, as the SNAP-25 and Syntaxin-1a/HPC1 antibodies only immunolabeled guinea pig horizontal cells in the outer retina (Lee and Brecha, 2010). Furthermore, photoreceptors express a different syntaxin isoform, syntaxin-3b, in their synaptic terminals (Morgans et al., 1996; Curtis et al., 2008; Hays et al., 2020).

**Figure 8:**
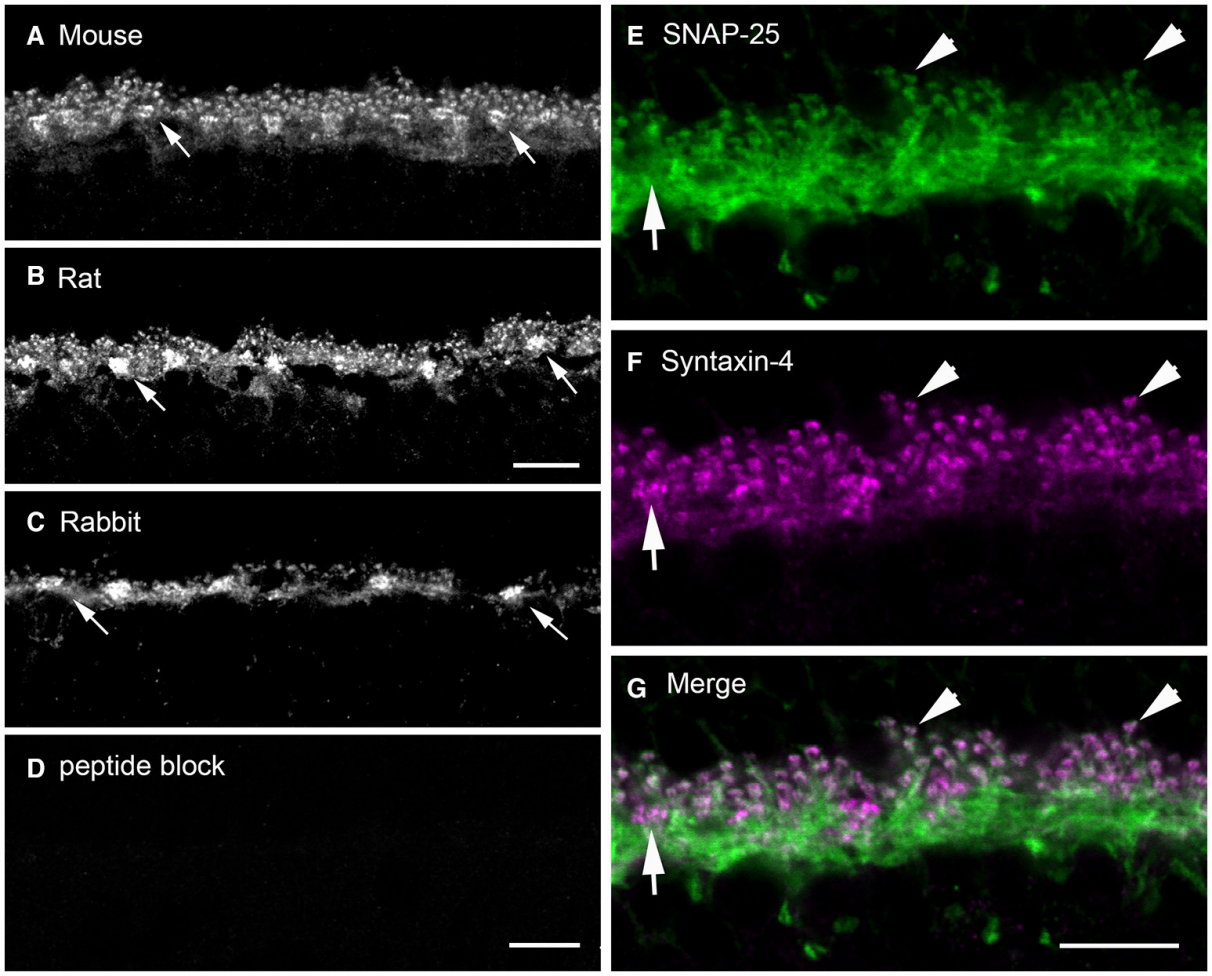
Syntaxin-4 immunolabeling is present in the outer plexiform layer of mouse, rat, and rabbit retinas and colocalizes with that of SNAP-25. **(A–C)** Localization of syntaxin-4 immunoreactivity in vertical sections of **(A)** mouse, **(B)** rat, and **(C)** rabbit outer retina. Note the prominent immunoreactivity in the OPL of all three species. Arrows point toward thickenings or sandwiches of syntaxin-4 immunolabeling. **(D)** Pre-adsorption of the antibody with the antigenic peptide abolishes specific labeling in rabbit retina. **(E–G)** Syntaxin-4 **(F)** immunolabeling co-localized with that of SNAP-25 in horizontal cell processes and endings **(E)** as seen in the **(G)** merged image in mouse retina. Arrows point to horizontal cell dendritic contacts with cone pedicles. Arrowheads point to immunolabeling in horizontal cell axonal endings. **(A,C,D)** Maximum intensity projection of 3 images, *z* = 0.6 μm. **(B)** Maximum intensity projection of 5 images, *z* = 0.46 μm. **(E–G)** Maximum intensity projection of 3 images, *z* = 0.6 μm. ONL, outer nuclear layer; OPL, outer plexiform layer; INL, inner nuclear layer. Scale bars, 10 μm (**A,B** in **B**, **C,D** in **D**, **E–G** in **G**). (Modified from Hirano et al., 2007).

We found syntaxin-4, another isoform that targets vesicles to the plasma membrane (Teng et al., 2001) is highly expressed in horizontal cells at axonal terminals and dendrites (Figure 8), where it is concentrated beneath cone pedicles (Figure 8, arrows), and in the lateral elements at photoreceptor terminals (Hirano et al., 2007). Figures 8A–C shows syntaxin-4 immunolabeling in the OPL of mouse (**A**), rat (**B**), and rabbit (**C**) retina, which co-localizes with the horizontal cell marker, calbindin (Hirano et al., 2007). Syntaxin-4 co-localizes with SNAP-25 in the endings of horizontal cells (Figures 8E–G, Hirano et al., 2007). Immunoelectron microscopy places syntaxin-4 immunoreactivity in the lateral elements at photoreceptor synapses (Figures 6C,D). In other neuronal systems, syntaxin-4 is found in postsynaptic membranes and marks a domain for ionotropic glutamate receptor exocytosis in dendritic spines in hippocampus (Kennedy et al., 2010; Bin et al., 2019) and NGF release from Schwann cells (Lin et al., 2017). At the *Drosophila* neuromuscular junction, syntaxin-4 is postsynaptic and is involved in retrograde signaling to motoneurons (Harris et al., 2016) to regulate neurotransmitter release and the number of presynaptic active zones and Ca channels (Harris et al., 2018).

**Figure 9:**
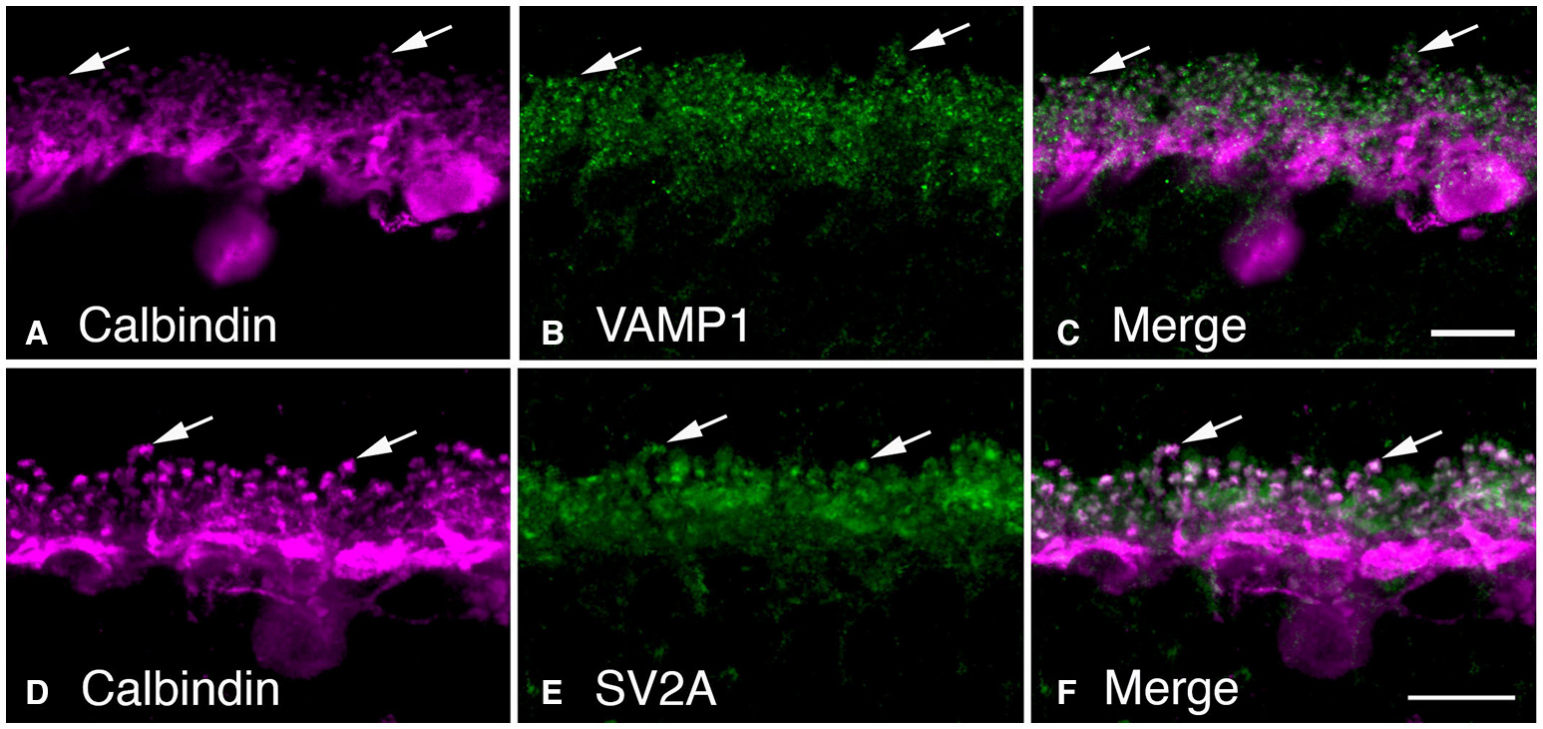
Synaptic vesicle proteins, VAMP-1 and SV2A, are found in horizontal cell synaptic endings. **(A–C)** VAMP-1 immunolabeling (**B**, green) was observed in the OPL of mouse retina. **(B)** Horizontal cells labeled with calbindin antibodies (**A**, magenta). **(C)** Merged image demonstrated the co-localization of VAMP-1 and calbindin immunoreactivities in horizontal cell processes and particularly in the synaptic endings (arrows). **(D–F)** Synaptic vesicle protein SV2A immunolabeling **(E)** was present in the OPL as large puncta. **(D)** Horizontal cells identified by calbindin immunoreactivity (magenta). **(F)** Merged image shows co-localization of SVA and calbindin immunoreactivities in horizontal cell endings. There is also SV2A immunolabeling surrounding the horizontal cell endings, in likely photoreceptor terminals. Maximum intensity projections, **A–C**, *z* = 0.6 μm; **D–F**, *z* = 6.42 μm. (Modified from Bitzer and Brecha, 2006; Brecha et al., 2010).

We observed VAMP-1, rather than VAMP-2, in horizontal cell endings by double label immunohistochemistry (Figures 9A–C, (Bitzer and Brecha, 2006; Lee and Brecha, 2009). VAMP-2 is the more common VAMP/synaptobrevin isoform in SNARE complexes at conventional synapses, with VAMP-1 occurring to a lesser degree (Elferink et al., 1989; Brunger et al., 2019). In well-fixed mouse retina, the VAMP-1 labeling was reported to be weaker than that of VAMP-2, in the plexiform layers (Sherry et al., 2003). The strong fixation may have resulted in difficulties in interpretation of VAMP-1 immunostaining, as VAMP-1 immunoreactivity did not appear to label synaptic structures (Sherry et al., 2003).

### Synaptic Vesicle Proteins

Given the prevalent view at the time that there were few or no synaptic vesicles in the horizontal cell endings [(Schwartz, 2002), but see (Dowling and Boycott, 1966; Dowling, 1970; Raviola and Gilula, 1975; Spiwoks-Becker et al., 2001; Zampighi et al., 2011)] we checked whether there were other key synaptic vesicle proteins in addition to VGAT. There are at least 40 different families of vesicle and synaptic proteins, including the synaptotagmins, synapsins, GTP-binding Rab proteins and complexins, that have critical roles in Ca^2+^-dependent transmitter release, including Ca^2+^ sensing, vesicle trafficking, and vesicle fusion (Jahn and Scheller, 2006; Takamori et al., 2006). Most of these proteins have multiple isoforms that are differentially expressed in the nervous system (Linial, 1997; Hong, 2005). From this screen, we localized several synaptic vesicle proteins to horizontal cell endings (Hirano et al., 2005, 2007, 2011; Lee and Brecha, 2010), supporting the hypothesis that horizontal cells contain synaptic vesicles, and transmitter is released by a vesicular mechanism.

SV2A is a ubiquitous synaptic vesicle transporter protein in the brain (Buckley and Kelly, 1985; Bajjalieh et al., 1992; Feany et al., 1992; Janz and Südhof, 1999) and is involved in sensing presynaptic calcium levels to prime synaptic vesicles for calcium-dependent exocytosis (Janz et al., 1999; Chang and Südhof, 2009; Wan et al., 2010). Knockout of SV2A resulted in a reduction in hippocampal GABAergic neurotransmission (Crowder et al., 1999). In outer retina, SV2A co-localized with VGAT in horizontal cell endings in likely synaptic vesicles (Lee and Brecha, 2010). Figures 9D–F shows SV2A co-localized with calbindin in horizontal cell endings, as well as in photoreceptor terminals (Brecha et al., 2010). SV2A was reported earlier to be transiently expressed in horizontal cells and cone photoreceptors during mouse retina development, but not in adult retina (Wang et al., 2003). The lack of double labeling for calbindin to clearly identify horizontal cell processes in the OPL in the relatively low-power magnification images makes it difficult to rule out horizontal cell labeling. In well-fixed adult mouse retina, SV2A was reported to be in cone ribbon synapses and a subset of conventional synapses; whereas, SV2B was in photoreceptor and bipolar cell ribbon synapses and SV2C, to sparse conventional synapses in the outer retina and starburst amacrine cells (Wang et al., 2003).

**Figure 10:**
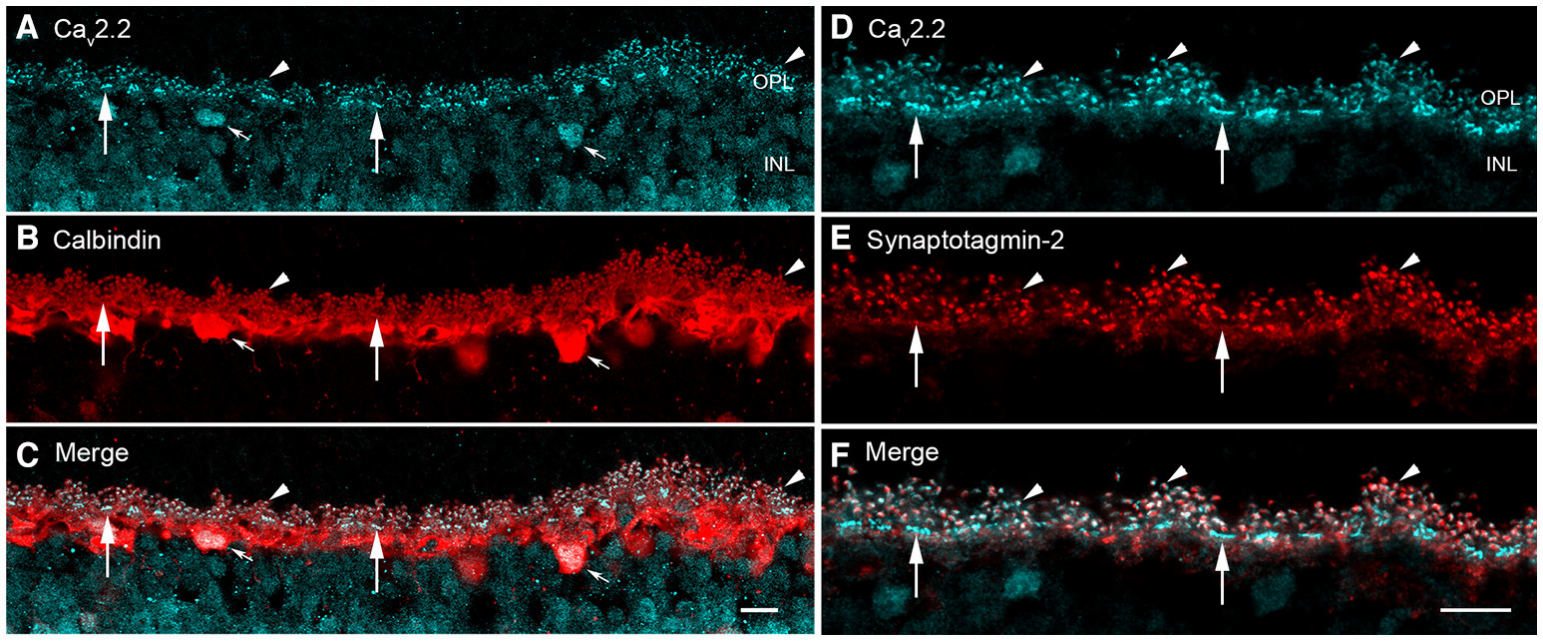
Voltage-gated calcium channels and calcium sensor synaptotagmin-2 are expressed in mouse horizontal cell endings. **(A–C)** Ca_v_2.2 (α1B, N-type) Ca channels immunolabeling (**A**, blue) occurred as discrete puncta in the OPL in mouse retina (arrowheads). **(B)** Calbindin immunolabeling (red) identifies horizontal cells in outer retina (arrowheads). **(C)** Merged image showed co-localization of Ca_v_2.2 and calbindin immunoreactivities (arrowheads), suggesting Ca_v_2.2 is at horizontal cell synaptic endings. Small arrows point to Ca_v_2.2 immunoreactivity in horizontal cell bodies, suggesting that Ca_v_2.2 is expressed by horizontal cells and not photoreceptors. **(D–F)** Ca_v_2.2 co-localizes with the calcium sensor synaptotagmin-2 in horizontal cells. **(D)** Ca_v_2.2 (blue) immunolabeling occurred as puncta (arrowheads) and bars (arrows) in the OPL. **(E)** Calcium sensor synaptotagmin-2 (red) immunolabeling occurred in horizontal cell processes and is concentrated in the tips. **(F)** Merged image showed that Ca_v_2.2 and synaptotagmin-2 are present in the same subcellular compartment of the horizontal cell axonal endings. The synaptotagmin-2 immunostaining in horizontal cell dendrites at cones (arrows) appeared to be less intense than at the axonal endings, perhaps simply reflecting the volume of the compartment. Maximum intensity projections, **A–F**, *z* = 1.20 μm. Scale bars, 10 μm (**A–C** in **C**, **D–F** in **F**). (Modified Hirano and Brecha, 2010).

Synaptotagmins form a complex with SV2 proteins in a calcium-dependent manner, in part to regulate presynaptic calcium levels (Marqueze et al., 2000; Südhof, 2002; Wan et al., 2010) and accelerate synaptic vesicle priming and initiate fast, calcium-triggered release (Südhof, 2013). Synaptotagmin-1 and−2 are synaptic vesicle proteins with two calcium binding motifs (C2A and C2B) involved in calcium sensing in calcium-triggered transmitter release (Littleton et al., 1993; Südhof and Rizo, 1996; Südhof, 2002; Grassmeyer et al., 2019). Synaptotagmin-2, but not synaptotagmin-1, is enriched in the horizontal cell endings at both rod and cone photoreceptor terminals in mouse, rat, and guinea pig retina (Fox and Sanes, 2007; Lee and Brecha, 2010). Figures 10D–F show the co-localization of synaptotagmin-2 with Ca_v_2.2, the principal, pore-forming subunit of N-type Ca channels (Hirano and Brecha, 2010). In the cerebellum, synaptotagmin-2 is the fast Ca sensor at the basket cell-Purkinje cell synapse (Chen et al., 2017).

Complexins interact with synaptotagmins and the SNARE complex in a calcium-dependent manner to regulate synchronous transmitter release (Rizo and Xu, 2015; Mortensen et al., 2016). In retina, complexin isoforms are differentially expressed (Hirano et al., 2005; Reim et al., 2005; Lee and Brecha, 2010) with complexin-1/2 found at conventional synapses and complexin-3 and−4 at ribbon synapses (Reim et al., 2005; Vaithianathan et al., 2013; Babai et al., 2016; Mortensen et al., 2016; Bhoi et al., 2020). Complexin-3 is also found at glycinergic synapses in the lobular appendages of AII amacrine cells (Landgraf et al., 2012). Complexin-1/2 localized to rabbit (Figure 6F), mouse and guinea pig horizontal cell endings (Hirano et al., 2005; Reim et al., 2005; Lee and Brecha, 2010) and GABAergic amacrine cells (Hirano et al., 2005; Reim et al., 2005). In addition to interacting with synaptotagmins, complexins bind to SNARE proteins to regulate SNARE complex assembly (Chen et al., 2002; Kummel et al., 2011; Li et al., 2011) to promote synchronous release from a readily releasable pool and to inhibit asynchronous release (Trimbuch and Rosenmund, 2016; Zhou et al., 2017).

Synapsins are a family of 4 abundant synaptic vesicle-associated phosphoproteins that regulate synaptic vesicle availability (Hilfiker et al., 1999) and are markers of conventional synapses in retina, but not of ribbon synapses (Mandell et al., 1990). Synapsin I was expressed at low levels in rabbit horizontal cells (Hirano et al., 2005), consistent with the likely horizontal cell labeling in ferret retina (Karne et al., 1997) and guinea pig horizontal cells, which show strong immunolabeling (Lee and Brecha, 2010). Ultrastructurally, synapsin I immunolabeling was found in the horizontal cell axonal endings at rod photoreceptor synapses (Figure 6E, Hirano et al., 2005). Consistent with the immunolabeling, synapsin mRNA localized to presumed horizonal cells in developing rat retina (Haas et al., 1990).

**Figure 11:**
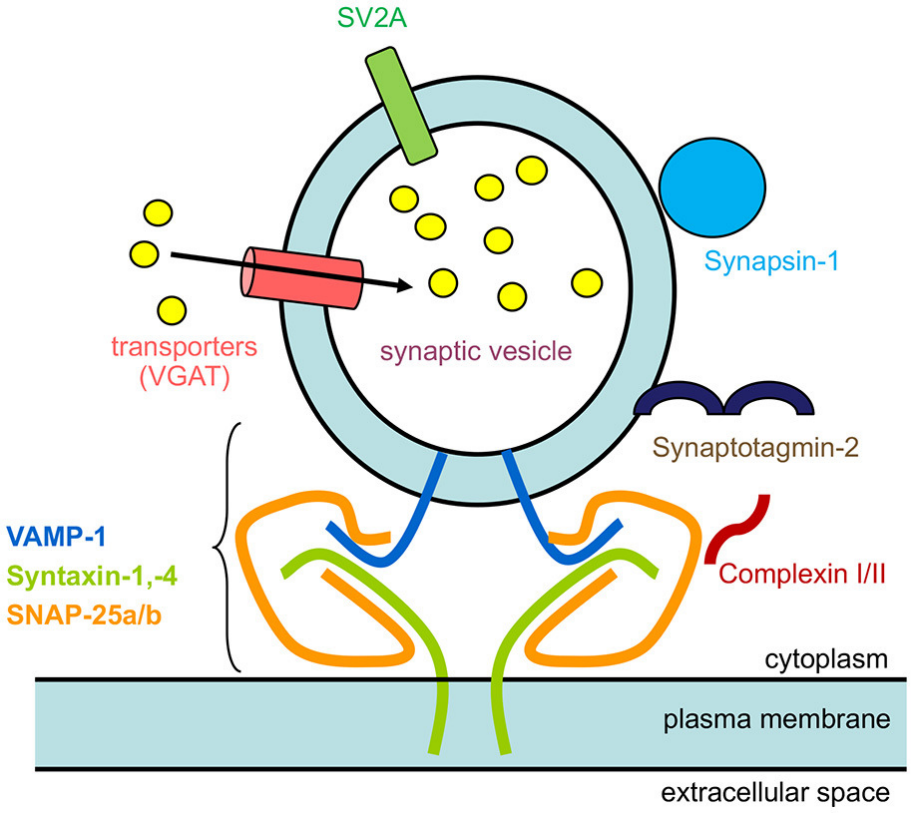
Schematic of synaptic proteins found in mammalian horizontal cells. The diagram depicts a synaptic vesicle studded with synaptic vesicle proteins, VGAT, a neurotransmitter transporter, SV2A, Synaptotagmin-2, a calcium sensor, Synapsin I, and SNARE protein, VAMP-1. The other 2 SNARE proteins that form the minimal complex are Syntaxin-1,−4, and SNAP-25, that brings the synaptic vesicle close to the plasma membrane for fusion. Finally, complexin-1/2 is a SNARE-associated protein. The yellow circles represent GABA that is accumulated inside synaptic vesicles by VGAT.

The localization of numerous synaptic vesicle proteins to horizontal cell processes and endings, including VGAT (Haverkamp et al., 2000; Cueva et al., 2002; Jellali et al., 2002; Guo et al., 2010; Lee and Brecha, 2010; Hirano et al., 2011), complexin-1/2, synapsin I (Hirano et al., 2005), SV2A (Brecha et al., 2010), and synaptotagmin-2 (Fox and Sanes, 2007; Lee and Brecha, 2010), and SNARE proteins (SNAP-25a/b, syntaxin-1a,−4, VAMP1) (Hirano et al., 2005, 2007, 2011; Bitzer and Brecha, 2006) is consistent with the idea that synaptic vesicles are present in horizontal cells and participate in calcium-triggered exocytosis (Figure 11).

### Presence of a Ca^2+^ Sensor and Voltage-Gated Ca Channels

The localization of synaptotagmin-2 to horizontal cells indicated that a calcium sensor for neurotransmitter release is present in these terminals (Figures 10D–F, Fox and Sanes, 2007; Lee and Brecha, 2010). Rabbit, cat and mouse horizontal cells express L-type voltage-dependent Ca^2+^ channels (Ueda et al., 1992; Löhrke and Hofmann, 1994; Schubert et al., 2006; Liu et al., 2016), which are known to regulate sustained transmitter release in photoreceptor and bipolar cells and to modulate transmitter release smoothly and continuously with changes in membrane potential that accompany changing levels of illumination (Corey et al., 1984; Wilkinson and Barnes, 1996; de la Villa et al., 1998; Barnes and Kelly, 2002; Morgans et al., 2005; Mercer and Thoreson, 2011; Van Hook et al., 2019). The minimal voltage-dependent inactivation, characteristic of L-type Ca^2+^ channels, is well-suited for maintaining constant output at these tonic synapses (Juusola et al., 1996). Figures 10A–C shows immunolabeling for Ca_v_2.2 and horizontal cell marker calbindin (Hirano and Brecha, 2010), and the co-localization of Ca_v_2.2 to the horizontal cell axonal terminals and at cone pedicle dendritic contacts suggest N-type Ca channels may play a role in transmitter release. In rat, Ca_v_1.2 (L-type, α1C), Ca_v_2.1 (P/Q-type, α1A), and Ca_y_2.2 (N-type, α1B) were localized by immunohistochemistry to horizontal cell endings (Liu et al., 2013). These findings are consistent with the physiological data supporting three types of voltage-gated Ca channels in mouse horizontal cells based on pharmacological discrimination using nifedipine/verapamil, ω-agatoxin IVA and ω-conotoxin GVIA, respectively (Schubert et al., 2006; Liu et al., 2013).

## Presence of Vesicles In Horizontal Cell Processes and Endings as Potential Vesicular Sources of GABA Release

**Figure 12:**
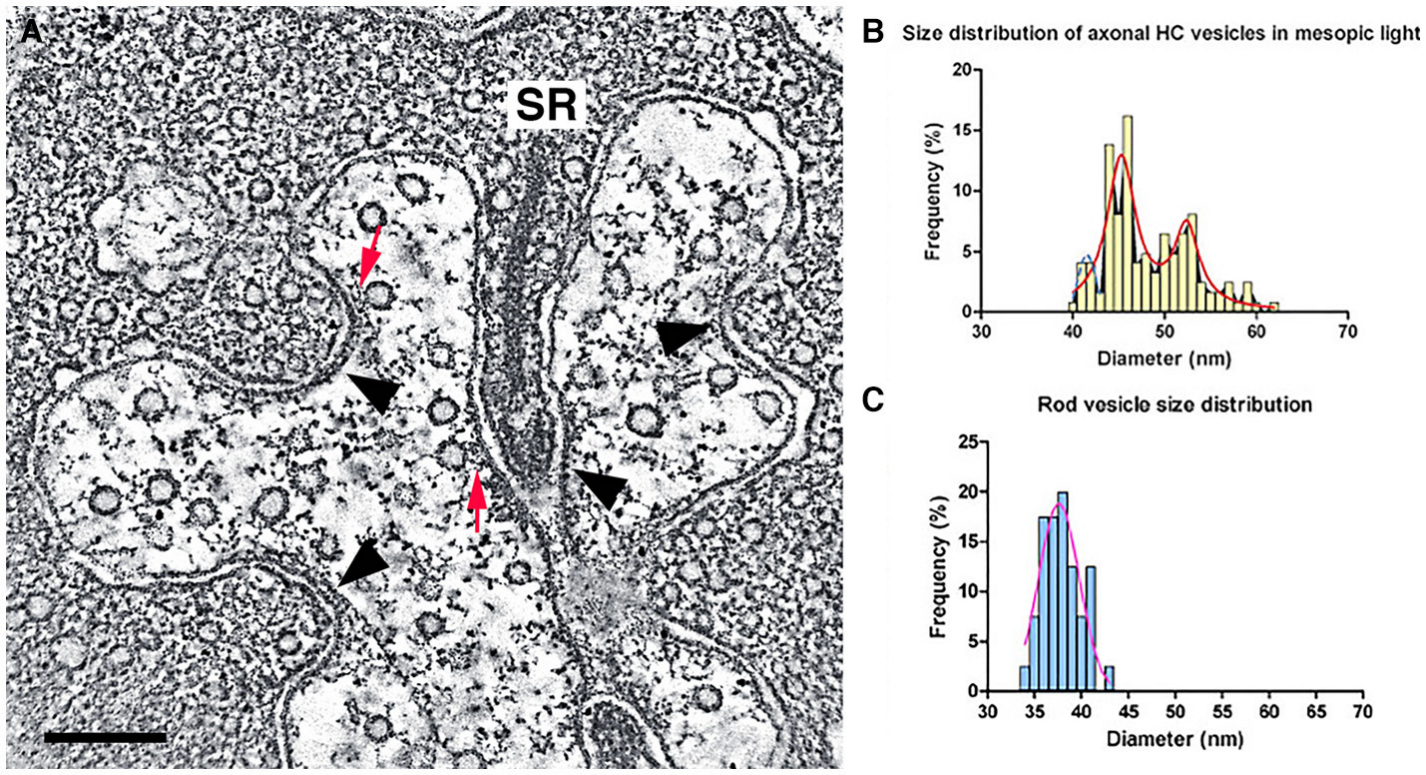
Small, clear-core vesicles are present in horizontal cell endings in rod spherules and cone pedicles. **(A)** A mouse rod photoreceptor spherule with a horizontal cell axonal terminal near the photoreceptor synaptic ribbon (SR) containing numerous small, clear-core vesicles. Clear-core vesicles represent small neurotransmitter-containing synaptic vesicles. The vesicles show fine fibrils extending from their membrane and in some examples, the vesicles are tethered to the plasma membrane or to plasma membrane specializations (red arrows). Plasma membrane specializations (arrowheads) are seen at infoldings of the horizontal cell endings and near the base of the synaptic ribbon. **(B,C)** Distribution of vesicle diameters in horizontal cell axonal endings **(B)** and in rod terminals **(C)**. Horizontal cell axonal vesicle diameters have a bimodal distribution, and overall horizontal cell axonal vesicle diameters are larger than rod photoreceptor vesicle diameters. **(A)** z-section (orthoslice) of a tomogram. Scale bar, 200 nm. (Modified from Brecha et al., 2010; Zampighi et al., 2011).

Initial electron microscopic studies of horizontal cell endings of cat, rabbit, and primate retina (Dowling and Boycott, 1966; Dowling et al., 1966; Raviola and Gilula, 1975) reported infrequent small, clear-core vesicles using different fixation protocols, with the most detailed report in the rat retina (Gray and Pease, 1971). Clear-core vesicles represent a type typically containing small molecule transmitters, such as GABA, glutamate, or acetylcholine, and not catecholamines or peptides. These vesicles are similar in appearance to the small, clear-core vesicles in adjacent photoreceptor terminals (Figure 12A).

We have used conical tomography electron microscopy (Zampighi et al., 2008, 2011) to evaluate horizontal cell dendritic and axonal endings in mouse and guinea pig photoreceptor invaginations. Conical electron microscopy is a high resolution, electron microscopic technique with ~3 nm isotropic resolution in the x-, y-, and z-planes. Essentially, this resolution eliminates the projection artifact common in thicker conventional and scanning block-face electron microscopic images that obscures fine cytoplasmic and membrane detail (Zampighi et al., 2008).

**Figure 13:**
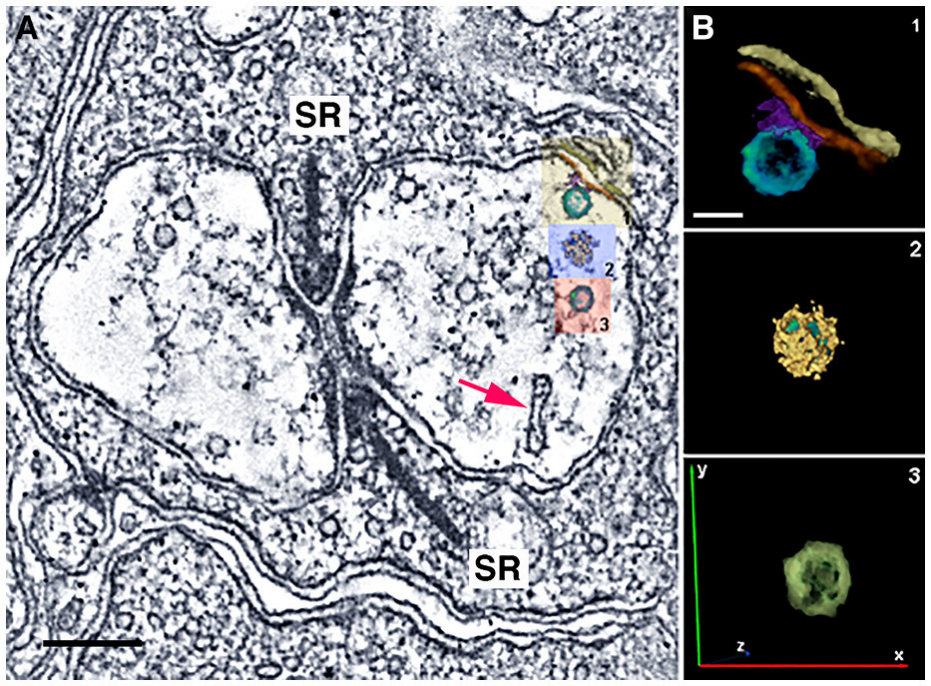
Horizontal cell endings contain different vesicle types. **(A)** A rod photoreceptor spherule with a horizontal cell axonal terminal with serial reconstructions of selected vesicles in mouse. Small vesicles with a conventional appearance are distributed throughout the terminal. A red arrow points to an endosome. In the right-side horizontal cell terminal, there is a small vesicle tethered to the plasma membrane (yellow box, **B1**). **(B)** Reconstructions of vesicles: **(B1)**, A small vesicle (yellow box in **A**, turquoise vesicle) that is likely attached to the horizontal cell plasma membrane (red) via synaptic proteins (purple). Other structures include the horizontal cell plasma membrane (red) and the rod plasma membrane (yellow-brown). **(B2)**: A clathrin-coated vesicle (blue box in **A**) in the cytoplasm. Clathrin cage (yellow); vesicle (green). **(B3)**: A small vesicle (red box in **A**, green) in the cytoplasm. **(A)** z-section (orthoslice) of a tomogram; **(B)** Reconstructions of 3 vesicles. Scale bar, 200 nm in **A**, 40 nm in **B**. (Modified from Zampighi et al., 2011).

We have identified numerous small, clear-core vesicles, clathrin-coated vesicles, and patches of plasma membrane thickenings with prominent cytoplasmic specializations in the mouse horizontal cell terminals (Figures 12A, 13A, Zampighi et al., 2011). The small, clear-core vesicles have several fine fibrils that are readily seen in the conical tomograms, although they are not seen in conventional electron micrographs. These vesicles are similar in appearance to descriptions of synaptic vesicles in neurons (Peters et al., 1991). A preliminary comparison in mouse horizontal cells indicates a greater number of vesicles in axonal endings compared to dendritic endings. Vesicle diameters in these endings range between 37 and 62 nm with 2 major peaks at 46 and 53 nm, and a smaller peak at 40–41 nm (Brecha et al., 2010). Overall, horizontal cell vesicle size is larger than the rod vesicle size (Figures 12B,C; *N* = 120; 6 endings). Interestingly, inspection of vesicle sizes in a primate cone terminal and adjacent horizontal cell dendrite (Raviola and Gilula, 1975) also shows that the vesicles in the cone cytoplasm are smaller overall than the vesicles in the horizontal cell dendritic ending and similarly in horizontal cell axon terminals (Moser et al., 2020). In addition, to numerous small vesicles, the horizontal cell terminal occasionally contained endocytotic (Figure 13A, red arrow) and clathrin-coated vesicles (Figure 13B2, Zampighi et al., 2011). Some larger and irregular shaped vesicles were also seen in horizontal cell terminals of rat or guinea pig retina (Gray and Pease, 1971). The presence of both endosomes (Figure 13A, red arrow) and clathrin-coated vesicles is indicative of active processes occurring in these terminals.

Horizontal cell membranes that are opposite and flanking the arciform density of the mouse photoreceptor terminal are characterized by membrane specialization in conventional electron microscopic preparations (Dowling and Boycott, 1966; Gray and Pease, 1971; Raviola and Gilula, 1975; Linberg and Fisher, 1988). Plasma membrane specializations (arrowheads) also occur along different infoldings of the horizontal cell plasma membrane within the invagination (Figure 12A, Zampighi et al., 2011). There are examples of small vesicles connected by thin tethers to the plasma membrane or are closely associated with these plasma membrane specializations (Figure 12A arrowheads, Figure 13A). In addition, small vesicles are near and adjacent to the plasma membrane in different parts of the horizontal cell terminal (Figures 12A, 13A). Together, these observations suggest the possibility that vesicle fusion and transmitter release sites are located at multiple sites within the horizontal cell terminals.

Vesicle clustering at membrane thickenings typical of many neuronal central synapses was not observed in early reports on primate, cat, rabbit, and rat horizontal cells (Dowling and Boycott, 1966; Raviola and Gilula, 1975; Kolb, 1977; Schaeffer et al., 1982; Peters et al., 1991). These findings may reflect a sampling issue of synapses that are sparsely distributed, as other ultrastructural studies on cat, rabbit, mouse, primate, mudpuppy, salamander, catfish, and turtle retinas demonstrated small clusters of synaptic vesicles in horizontal cell processes adjacent to membrane thickenings in bipolar cell dendrites, suggestive of horizontal cells feedforward synapses (Dowling et al., 1966; Olney, 1968; Dowling and Werblin, 1969; Dowling, 1970; Lasansky, 1973; Fisher and Boycott, 1974; Raviola and Gilula, 1975; Kolb and Jones, 1984; Sakai and Naka, 1986; Linberg and Fisher, 1988; Greferath et al., 1994). In human retina, horizontal cells were shown to make synaptic contacts with rod bipolar cell dendrites and the rod spherule within the invagination (Linberg and Fisher, 1988). Infrequent horizontal cell synapses with interplexiform processes were found in cat and rabbit also (Kolb, 1974; Kolb and West, 1977; Greferath et al., 1994).

The relative dearth and scattered distribution of synaptic vesicles in horizontal cell endings are similar to the observations of dopaminergic neurons that signal by extrasynaptic somatodendritic release, where it has been difficult to unequivocally identify the organelles (small clear-core vesicles, tuberovesicles, and large dense-core vesicles) that mediate dopamine release (Puopolo et al., 2001; Fortin et al., 2006; Hirasawa et al., 2012, 2015; Ludwig et al., 2016). Moreover, the dopaminergic amacrine cell perikaryon does not contain active zones; although, active zones were observed at their dendritic synapses with AII amacrine cells (Puopolo et al., 2001).

## Depolarization- and Calcium-Dependent Synaptic Vesicle Fusion and Recycling

Ca^2+^-regulated transmitter release is a well-established mechanism in the CNS (Südhof, 2013; Kaeser and Regehr, 2014; Rizo, 2018). In the mammalian retina, evidence supports the idea that horizontal cell transmitter release is regulated by Ca^2+^. The support includes demonstration of voltage-gated Ca^2+^ currents (I_Ca_) in horizontal cells (Schubert et al., 2006; Liu et al., 2016) and the localization of L-, N-, and P/Q-type Ca^2+^ channels (Liu et al., 2013) and the Ca^2+^ sensor, synaptotagmin-2 (Figures 10D–F, Hirano and Brecha, 2010; Lee and Brecha, 2010) to horizontal cell terminals. N-type Ca^2+^ channels are of particular interest, since they mediate vesicle release at central synapses (Catterall, 2011). Somatodendritic secretion of dopamine and peptides relies on L-type Ca channels primarily (Ludwig et al., 2016). In striatum, dopamine release can involve N-, Q-, T-, and L-type voltage-gated Ca channels, depending on neuronal activity, diverse calcium dependence, and calcium buffering in different cellular domains (Brimblecombe et al., 2015).

**Figure 14:**
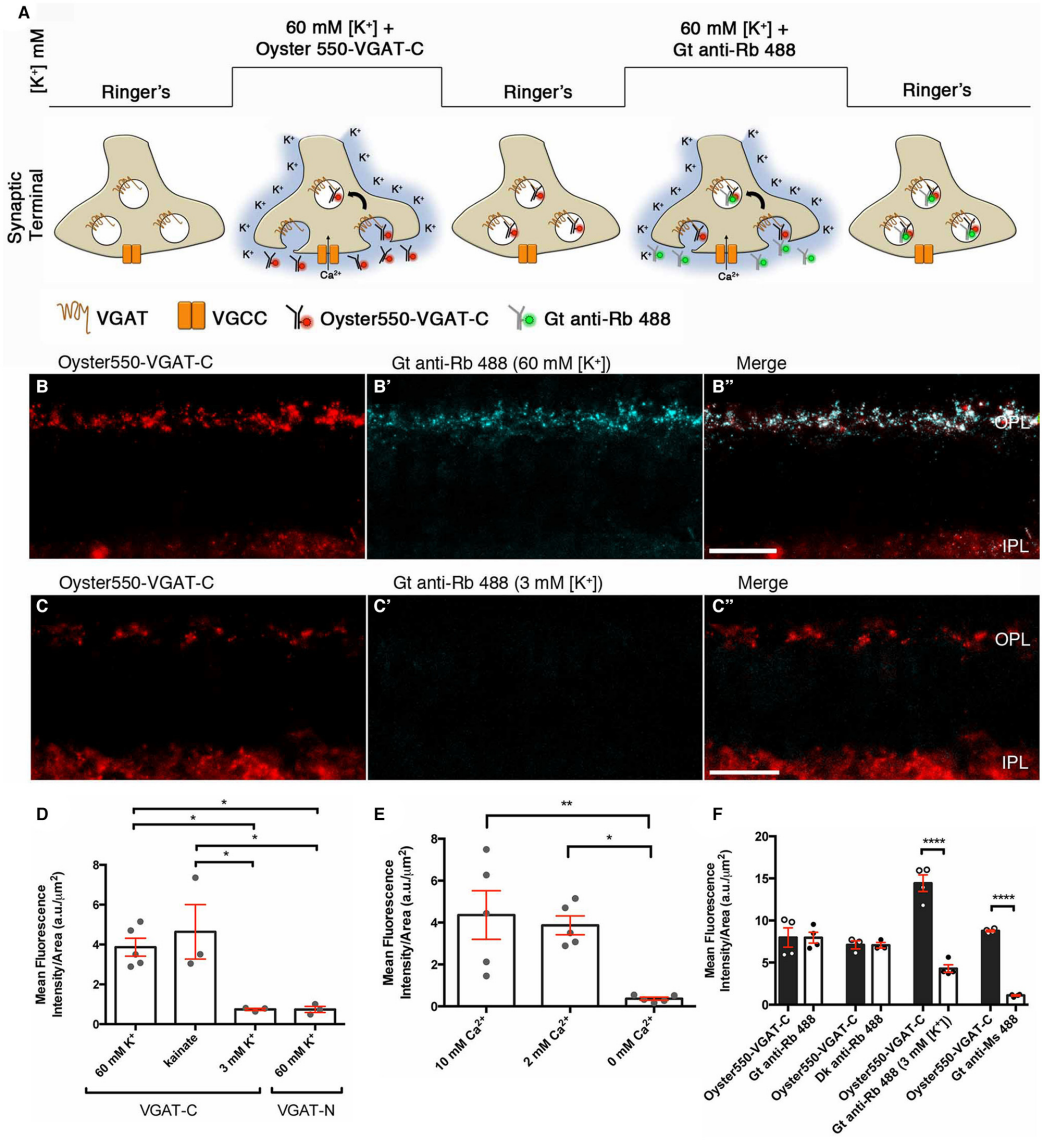
A VGAT-C synaptic vesicle fusion assay demonstrated that vesicle fusion occurs in a depolarization- and calcium-dependent manner in horizontal cell endings. **(A)** Schematic depicts the luminal VGAT-C vesicle fusion assay protocol. Retinal slices are infused with extracellular medium containing VGAT-C antibodies, then depolarized with high [K^+^]_o_ that activates voltage-gated calcium channels and the subsequent Ca^2+^ influx triggers synaptic vesicle fusion with the plasma membrane and, thus, exposure to the VGAT-C antibodies in the extracellular space. **(B,C)** panels showed the Oyster550-VGAT-C immunolabeling (red) of the fused vesicles. **(B')** panels showed that a second round of depolarization and vesicle fusion labeled by Alexa488-conjugated secondary antibodies (blue) in the extracellular medium that recognizes the VGAT-C rabbit polyclonal antibodies from the first round of labeling, indicating that the synaptic vesicles recycle, as seen in the merged images (**B”**, white). **(C'-C”,D)** Control experiments showed that there was little to no vesicle fusion when slices were maintained in the basal 3 mM [K^+^]_o_. **(D)** Quantification of VGAT-C immunofluorescence was significantly increased when depolarized with high (60 mM) [K^+^]_o_ or 50 μM kainate, but not with basal 3 mM [K^+^]_o_. When VGAT-N antibodies that recognize a cytosolic epitope not exposed to extracellular milieu were used, no specific labeling of horizontal cell endings was observed. This finding indicated that the VGAT-C immunolabeling was not a result of non-specific uptake of antibody *p < 0.02. **(E)** Quantification of VGAT-C immunofluorescence under different extracellular Ca concentrations. Significant increases in VGAT-C immunolabeling were observed in basal (2 mM) and high (10 mM) [Ca^2+^]_o_ conditions. In contrast, little to no labeling occurred in nominally calcium-free media (0 mM) *p < 0.01, **p < 0.005. **(F)** Quantification of VGAT-C immunofluorescence during multiple rounds of depolarization, marked by different fluorophores (Oyster550 vs. Alexa488). Goat or donkey anti-rabbit IgG-Alexa488 recognized the VGAT-C primary antibody from the initial round of immunolabeling. In contrast when goat anti-rabbit-Alexa488 IgG was present during the subsequent incubation period in basal [K^+^]_o_, significantly less immunolabeling was observed. Similarly, when a goat anti-mouse IgG-Alexa488 was used, little to no immunolabeling was observed ****p < 0.001. **(B,B',B”,C,C',C**,”**)** maximum intensity projections, *z* = 5.0 μm. Scale bars, 20 μm. (Modified from Lee, 2010; Vuong et al., 2011).

Using a luminal VGAT-C antibody in a retinal slice assay, we show that the voltage-gated Ca channels participate in Ca^2+^-mediated vesicular release from horizontal cells Figure 14A. We developed a retinal slice assay (Lee, 2010; Vuong et al., 2011) to monitor VGAT-expressing vesicles, based on topological studies that showed the C-terminus of VGAT is located within the vesicle lumen and using a fluorophore-conjugated, C-terminal directed VGAT (VGAT-C) antibody (Martens et al., 2008). Depolarization resulted in an Oyster550-VGAT-C terminus antibody labeling of the internal face of exocytosed synaptic vesicles, now exposed to the extracellular milieu containing the Oyster550-VGAT-C antibod (Figure 14A). In retinal slices, depolarization with high [K^+^] or 50 μM kainate (Figure 14D) in the presence of the VGAT-C antibody resulted in punctate VGAT-C labeling of horizontal cell endings in the OPL (Figures 14B,C), indicative of synaptic vesicles fusion with the plasma membrane. Vesicle fusion is only detected with the VGAT-C antibody and not with a N-terminal, cytoplasmically directed VGAT antibody (Figure 14D), indicating the labeling was not non-specific uptake. Labeling is absent or below detection in control experiments [e.g., basal 3 mM [K^+^] (Figures 14C',D), Oyster550-VGAT-N antibodies (Figure 14D)]. We showed the VGAT-C antibody uptake in horizontal cell processes occurred in basal 2 and 10 mM [Ca^2+^]_o_; whereas, no labeling occurred in nominally 0 mM [Ca^2+^]_o_ (Figure 14E, Supplementary Figure 1) or in the presence of general (Cd^2+^, Co^2+^) and voltage-gated Ca channel subtype-specific blockers (ω-agatoxin, ω-conotoxin, nifedipine) (Supplementary Figure 2). These data indicate that the vesicle fusion in horizontal cell endings was depolarization- and calcium-dependent. Further, multiple rounds of labeling with depolarization, depicted in the schematic in Figure 14A, could be visualized using Alexa488-conjugated secondary antibodies to the VGAT-C primary antibodies (Figures 14B,B',B”,F), suggesting that the initially labeled vesicles are capable of recycling (Lee, 2010; Vuong et al., 2011).

## Functional Analysis of Horizontal Cell Signaling

### Feedback to Photoreceptors

**Figure 15:**
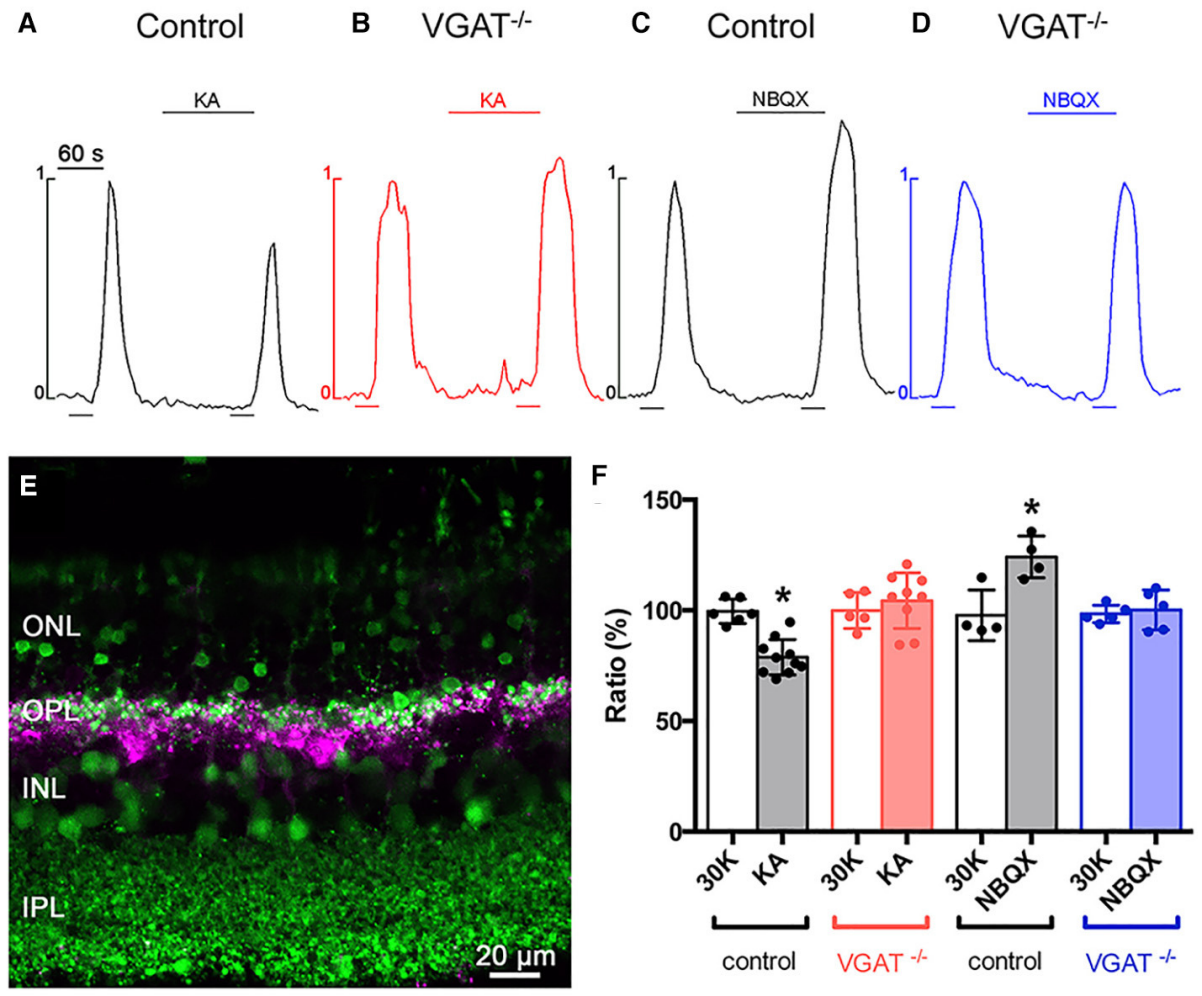
Deletion of VGAT in horizontal cells results in loss of inhibitory feedback modulation of photoreceptor [Ca^2+^]_i_. **(A–D)** Changes to the strength of feedback inhibition, which were increased by kainate and decreased by NBQX, were eliminated in the VGAT KO mice. Twin 30-s pulses (bars below traces) of high [K^+^]_o_ were applied to mouse retinal slices and, during the second pulse, either 50 μM kainate (bar above trace) was used to pharmacologically depolarize, or 50 μM NBQX to hyperpolarize, the horizontal cell membrane potential. In wild-type (VGAT^+/+^) retina, **(A)** kainate decreased the rise in photoreceptor [Ca^2+^]_i_ (black traces), suggesting an increase in feedback inhibition while, in *Cx57-VGAT*^−/−^ knockout retinal slices **(B)** kainate (red traces) produced no change. In *Cx57-VGAT*^+/+^ retina, **(C)** NBQX increased photoreceptor [Ca^2+^]_i_ (black traces), representing a reduction in feedback inhibition, whereas **(D)** NBQX (blue traces) did not change the photoreceptor [Ca^2+^]_i_ in Cx57-VGAT^−/−^ retina, suggesting elimination of feedback inhibition to photoreceptors. These results show that GABA release from horizontal cells is essential for feedback inhibition. **(E)** Confocal image of a *Cx57-tdTomato* retinal slice loaded with fluo-4 (green). tdTomato (magenta) identifies the horizontal cells. **(F)** Summary of photoreceptor Ca signal amplitudes in retinal slices from *VGAT*^+/+^ and *VGAT*^−/−^ mice treated with kainate and NBQX *p < 0.05. ONL, Outer nuclear layer; OPL, outer plexiform layer; INL, inner nuclear layer. (Modified from Hirano et al., 2016a).

Finally, we showed that feedback inhibition to photoreceptors occurs in a GABA-dependent manner to modulate the photoreceptor calcium current (Figure 15). To assay feedback, photoreceptors in slices (Figure 15E) were loaded with the calcium indicator Fluo-4 (green) in a *Cx57-tdTomato* retina, where the horizontal cells express the red fluorescent reporter tdTomato (converted to magenta), to show the relationship between horizontal cell processes and the photoreceptor cell bodies that were imaged. The increase in photoreceptor intracellular Ca^2+^ in response to pulses of 30 mM K^+^ was evaluated using drugs that depolarized or hyperpolarized horizontal cells (Vessey et al., 2005; Liu et al., 2013; Hirano et al., 2016a). A pulse of 30 mM K^+^ drove Ca influx through the voltage-gated calcium channels in the photoreceptors in control conditions, and then, when a second pulse was applied in the presence of kainate to depolarize the horizontal cells, the second pulse produced a smaller peak in intracellular Ca^2+^, showing that horizontal cell depolarization produced an inhibitory signal on the photoreceptor calcium channels (Figure 15A). Conversely, when the horizontal cells were hyperpolarized with 2,3-Dioxo-6-nitro-1,2,3,4,-tetrahydrobenzo[*f* ]quinoxaline-7-sulfonamide (NBQX), via blockade of ionotropic glutamate receptors during the second pulse, the calcium signal in photoreceptors is increased, indicating decreased feedback inhibition from horizontal cells to the photoreceptors (Figure 15C). These findings are consistent with reports in mouse retina (Babai and Thoreson, 2009). Kainate did not produce a change in photoreceptor calcium signal upon superfusion prior to the high-K^+^ pulse, consistent with a lack of ionotropic glutamate receptors on photoreceptors (Babai and Thoreson, 2009). To evaluate the role of vesicular GABA release in this feedback, we conditionally knocked out VGAT by crossing the horizontal cell-specific Cx57-iCre mouse (Hirano et al., 2016a) with a floxed VGAT mouse line (Tong et al., 2008). With the VGAT gene deleted, the neurotransmitter, most likely GABA, cannot be packaged into synaptic vesicles and released. Immunostaining for VGAT confirmed that the VGAT was selectively knocked out in horizontal cells (Hirano et al., 2016a). Whole-cell recordings of VGAT^−/−^ horizontal cells showed that the voltage-gated K^+^ and Ca^2+^ membrane currents were normal. In the horizontal cell VGAT knockout, kainate did not produce increased feedback inhibition and NBQX did not result in decreased feedback inhibition (Figures 15B,D,F, Hirano et al., 2016a). These data show that the loss of horizontal cell VGAT eliminated feedback inhibition onto photoreceptors.

## Discussion

In mammals, a preponderance of experimental findings indicates that retinal horizontal cells utilize a vesicular mechanism of transmitter release. The evidence for GABA as the horizontal cell neurotransmitter is the presence of GABA immunoreactivity and of GABA synthesizing enzymes (GAD65 and/or GAD67), and postsynaptic targets bearing GABA receptors (photoreceptors, bipolar cells) as well as autoreceptors on horizontal cells. Neonatal rabbit horizontal cells show ^3^H-GABA uptake that is downregulated after P5 (Redburn and Madtes, 1986); however, adult mammalian horizontal cells are atypical GABAergic neurons, in that they do not express plasmalemmal GABA transporters. The GABA transporters are expressed by Müller cells, whose processes ensheath photoreceptor synapses (Guo et al., 2009). The horizontal cells express SNARE proteins required for membrane fusion of synaptic vesicles (SNAP-25, VAMP-1, and syntaxin-1a & -4). The fusion of VGAT-bearing synaptic vesicles with the plasma membrane is depolarization- and calcium-dependent and show the capacity for multiple rounds of vesicle fusion in retinal slice preparations. The selective knockout of VGAT from horizontal cells that resulted in the loss of the tonic GABA current (Grove et al., 2019) and disrupted feedback inhibition to photoreceptors, showing that GABA release plays an integral role in these cells' neurotransmission (Hirano et al., 2016a).

The low numbers and the scattered appearance of synaptic vesicles in horizontal cell terminals as well as the absence of clearcut active zones (Figures 12, 13) are morphological features found in dendrites that are known to release transmitter; for example, somatodendritic dopamine and GABA release from dopaminergic neurons in the CNS (Hirasawa et al., 2015; Ludwig et al., 2016). In striatum, only a third of the dopaminergic boutons expressed a minimal active zone-like cluster of RIM, bassoon and ELKS (Liu et al., 2018). Ultrastructural analysis of dopaminergic amacrine cell somata revealed no active zones and few synaptic vesicles and tubulovesicular organelles (Puopolo et al., 2001). Nevertheless, somatodendritic release exhibits properties of regulated exocytosis, such as calcium dependence (Chen et al., 2006), the involvement of SNARE protein isoforms SNAP-25, VAMP2, and syntaxin 3b (Fortin et al., 2006; Witkovsky et al., 2009; Rice and Patel, 2015; Ludwig et al., 2016), voltage-gated Ca channels (Puopolo et al., 2001; Brimblecombe et al., 2015; Rice and Patel, 2015), and quantal release (Jaffe et al., 1998; Puopolo et al., 2001). The subtypes of voltage-gated Ca channels involved can differ in dopaminergic neurons and may reflect different modes of release (e.g., somatodendritic vs. axonal, firing patterns) (Ludwig et al., 2016). In retina, dopamine acts at synapses as well as by volume transmission (Witkovsky, 2004). The abundance of GABA_A_ receptors in the OPL along with the relatively few synapses found in horizontal cells to bipolar cell dendrites suggests that horizontal cell GABA may be acting by volume transmission. From the robust presence of syntaxin 4 and SNAP-25 throughout horizontal cell processes, it would be interesting to know if GABA release occurred extrasynaptically as well as synaptically from horizontal cells. Also, these SNARE proteins may function in the regulation of GABA or ionotropic glutamate receptor exocytosis, as syntaxin-4 is reported to be important for postsynaptic dendritic exocytosis in hippocampal neurons (Kennedy et al., 2010; Ovsepian and Dolly, 2011; Gu and Huganir, 2016; Bin et al., 2019).

Transmitter release by a regulated vesicular mechanism would be highly advantageous for fine control of feedback and feed-forward action in the outer retina, as there are multiple molecular control points to modulate secretion from horizontal cells that utilizes the bicarbonate permeability of GABA_A_ receptors to regulate cleft pH (Grove et al., 2019; Barnes et al., 2020). For instance, VGAT's dependence on a proton gradient for GABA uptake would influence vesicular GABA concentrations (Reimer et al., 1998) and, by extension, postsynaptic responses. The possible complex of VGAT and GAD65 (Wei and Wu, 2008) on synaptic vesicles in horizontal cell synaptic endings and its regulation on demand (Buddhala et al., 2009) would also stimulate GABA loading of synaptic vesicles. The highly regulated cascade of SNARE protein interactions in exocytosis would allow for a precise control of the rate and level of transmitter secretion. Local modulation of membrane potential at different endings could also differentially influence presynaptic Ca^2+^ channel dynamics and influence local GABA release.

The demonstration of GABA and its synthetic enzyme GAD65 and/or GAD67 in adult mammalian horizontal cells supports the notion that GABA is acting as a transmitter, despite not bearing GATs, notably like cerebellar Purkinje cells (Ribak et al., 1996; Guo et al., 2009). Müller cell processes that envelop photoreceptor terminals in the OPL are well placed to take up GABA (Guo et al., 2009), similar to Bergmann glia around the Purkinje cells (Ribak et al., 1996). The GABA receptors on horizontal cells and bipolar cell dendrites (Vardi and Sterling, 1994; Wässle et al., 1998; Haverkamp et al., 2000) indicate the receptor targets of the GABA released by horizontal cell are present.

The presence of GABA_A_ receptor ρ2 (Grove et al., 2019) immunolabeling on horizontal cell terminals implies a significant role for tonic GABA modulation of horizontal cell membrane potential and conductance, signaling which is mediated by graded regulation rather than phasic synaptic transmission. Horizontal cells appear to be the primary source of GABA in the outer retina, as VGAT knockout resulted in a loss of the TPMPA-sensitive GABA-induced current in horizontal cells and feedback regulation of photoreceptor Ca channels (Grove et al., 2019). In primate retina, Haverkamp and colleagues (Haverkamp et al., 2000; Puller et al., 2014) described layers of horizontal cell processes under primate cone pedicles and GABA_A_ receptor-bearing bipolar cell dendrites sandwiched between the two layers, and postulated that a GABA tone may be present. There is an enrichment of syntaxin-4 in horizontal cell processes at S-cones in primate retina and combined with expression of GABA_A_ receptor α1 and ρ subunits and the chloride-accumulating transporter NKCC vitreal to S-cone pedicles is suggestive of HII horizontal cell to blue cone bipolar cells feedforward signal transmission (Puller et al., 2014). There are also interplexiform processes from GABAergic tyrosine hydroxylase (TH) amacrine cells that form synapses in the OPL onto bipolar cell processes (Dowling and Ehinger, 1975; Kolb and West, 1977; Linberg and Fisher, 1986; Chun and Wässle, 1989; Greferath et al., 1994), as well as a non-dopamine containing GABAergic interplexiform cell in mouse (Dedek et al., 2009), that might contribute to GABA levels in the OPL (Chun and Wässle, 1989; Witkovsky et al., 2008). Grove et al. (2019) showed that the high-affinity, non-desensitizing GABA_A_ρ receptors on horizontal cell endings generate a tonic GABA current in the outer retina, most notably within the photoreceptor terminal synapse. Changes in tonic inhibition can alter neuronal and network properties, due to a persistent increase in input conductance that will regulate membrane excitability and alter the gain of a neuron's input-output relationship, and thus a neuron's responsiveness (Semyanov et al., 2004; Walker and Semyanov, 2008; Lee and Maguire, 2014).

In addition to feedback to photoreceptors, GABA_A_ receptors on bipolar cell dendrites relay the horizontal cell feedforward signal (Vardi et al., 1992; Vardi and Sterling, 1994; Enz et al., 1996; Wässle et al., 1998; Haverkamp et al., 2000; Hoon et al., 2015; Chaffiol et al., 2017), although which bipolar cell type and the GABA_A_ receptor subtypes used are not yet well-defined. The GABA_A_ receptors are ρ-containing or extrasynaptic GABA_A_ receptors containing δ subunits, such as those that contain GABA_A_ receptor α6 subunits, are high affinity and non-desensitizing, and mediate tonic inhibition in other CNS areas (Farrant and Nusser, 2005; Glykys and Mody, 2007; Belelli et al., 2009; Brickley and Mody, 2012). Consistent with higher levels of GABA in the interstitial space of the OPL, the background labeling for GABA was observed to be higher in the OPL (Chun and Wässle, 1989).

Conical electron tomography of mouse rod spherules and cone pedicles clearly demonstrate the presence of small, clear-core vesicles in horizontal cell axonal endings and dendritic endings, that are slightly larger compared to synaptic vesicles in rod and cone photoreceptors. Furthermore, the conical tomograms reveal putative active zones and membrane densities in the horizontal cell endings (Zampighi et al., 2011), endocytosis of clathrin-coated vesicles and vesicle tethers, indicative of vesicle specializations and vesicular activity that were not observed in thicker ultrathin sections due to projection artifacts. The presence of synaptic vesicles in horizontal cells is supported by conventional transmission electron micrographs from mouse (Spiwoks-Becker et al., 2001), cat and primates (Dowling and Boycott, 1966; Dowling, 1970; Raviola and Gilula, 1975; Linberg and Fisher, 1988), that depict small, clear-core vesicles in horizontal cell processes at both rod and cone photoreceptor synapses. The presence of synaptic vesicle proteins, such as VGAT and SV2A, in horizontal terminals further supports the conclusion of the presence of synaptic vesicles in these terminals.

These synaptic vesicles are the cellular substrate for the many synaptic vesicle proteins localized to horizontal cell endings, including VGAT, SV2A, synapsin I, complexin-1/2, synaptoporin (Brandstätter et al., 1996a; Hirano et al., 2005, 2007; Lee and Brecha, 2010). The SNARE complex proteins of syntaxin-1a and syntaxin-4, VAMP-1, and SNAP-25 (Hirano et al., 2005, 2007, 2011) along with the SNARE complex associated proteins (complexin-1/2 and synaptotagmin-2; Hirano et al., 2005) mediate and modulate vesicle fusion with the membrane. The use of less common isoforms, e.g. VAMP1, synaptotagmin-2, complexin-1/2, for synaptic vesicle release may reflect different kinetics and/or regulation at this tonic, graded potential GABAergic synapse (Reim et al., 2005; Hua et al., 2011; Chung and Raingo, 2013), similar to the usage of alternative synaptic protein isoforms at ribbon synapses (Moser et al., 2020). The subcellular localization of VAMP1 to horizontal cell terminals suggest it participates in synaptic transmission and that other VAMP isoforms may mediate vesicle trafficking between other cellular compartments within the cell. Although the precise role of syntaxin-4 remains to be determined, its high level of expression in horizontal cells, in addition to syntaxin-1a, likely reflect distinct pools of vesicles trafficked to the membrane. The calcium sensor, synaptotagmin-2, is preferentially expressed at cerebellar GABAergic synapses, where it is the fast Ca sensor and responsible for faster replenishment of the readily releasable pool, necessary for fast feedforward inhibition (Chen et al., 2017).

The functional VGAT-C antibody uptake studies indicated that the VGAT-containing synaptic vesicles fuse with the plasma membrane in a depolarization- and calcium-dependent manner, characteristic of vesicular exocytosis of transmitter (Südhof, 2013). Moreover, the synaptic vesicles recycle, as observed from the multiple rounds of labeling. Finally, the knockout of horizontal cell VGAT resulted in loss of GABA release and eliminated feedback inhibition of photoreceptor calcium channels (Hirano et al., 2016a; Grove et al., 2019). Together these findings show that the vesicular release of GABA from mammalian horizontal cells plays an essential role in horizontal cell synaptic transmission.

Grove et al. (2019) demonstrated that cone photoreceptor calcium currents are modulated by picrotoxin and TPMPA (see Grove et al., Figures 1–2), and that they act at GABA_A_R-ρ receptors on the horizontal cell endings (see Grove et al., Figures 2I–K), not photoreceptors. This GABAergic modulation is absent in the presence of HEPES, indicating pH sensitivity. Grove et al. (2019) extended the findings in Hirano et al. (2016a) by showing that GABA release by horizontal cells acts back onto its own GABA receptors, and that the GABA release (and cone Ca channel modulation) is abolished in *Cx57-VGAT*^−/−^ horizontal cells, concluding that the bicarbonate flux through these tonic GABA receptors regulates the synaptic cleft pH. The full hybrid GABA-pH model is much more detailed (see Figure 8, Grove et al., 2019; Figure 12, Barnes et al., 2020), including roles for sodium-proton exchangers (NHEs), the bicarbonate equilibrium potential and horizontal cell membrane potential excursions induced by GABA and glutamate (Grove et al., 2019; Barnes et al., 2020).

## Author Contributions

AH, SS, SB, and NB designed the experiments. HV and HK performed the VGAT-C functional labeling experiments. AH and HK performed the immunohistochemistry. HK performed PLA. CS performed the conical tomography. SB managed the calcium imaging experiments. AH, SB, and NB wrote the manuscript. All authors contributed to the article and approved the submitted version.

## Conflict of Interest

The authors declare that the research was conducted in the absence of any commercial or financial relationships that could be construed as a potential conflict of interest.
