# Supplementary material for: Vesicular Release of GABA by Mammalian Horizontal Cells Mediates Inhibitory Output to Photoreceptors
Source: Front Cell Neurosci. 2020 Dec 1;14:600777. doi: 10.3389/fncel.2020.600777 (PMC7735995; doi:10.3389/fncel.2020.600777)
Supplement: Supplementary file 1 [file Table_1.DOCX]

Hirano et al., Vesicular Release, Supplementary Material

**Figure S1. Basal [K^+^]_o_ shows minimal background VGAT-C uptake. VGAT-N with a cytoplasmic epitope does not label fused vesicles.**


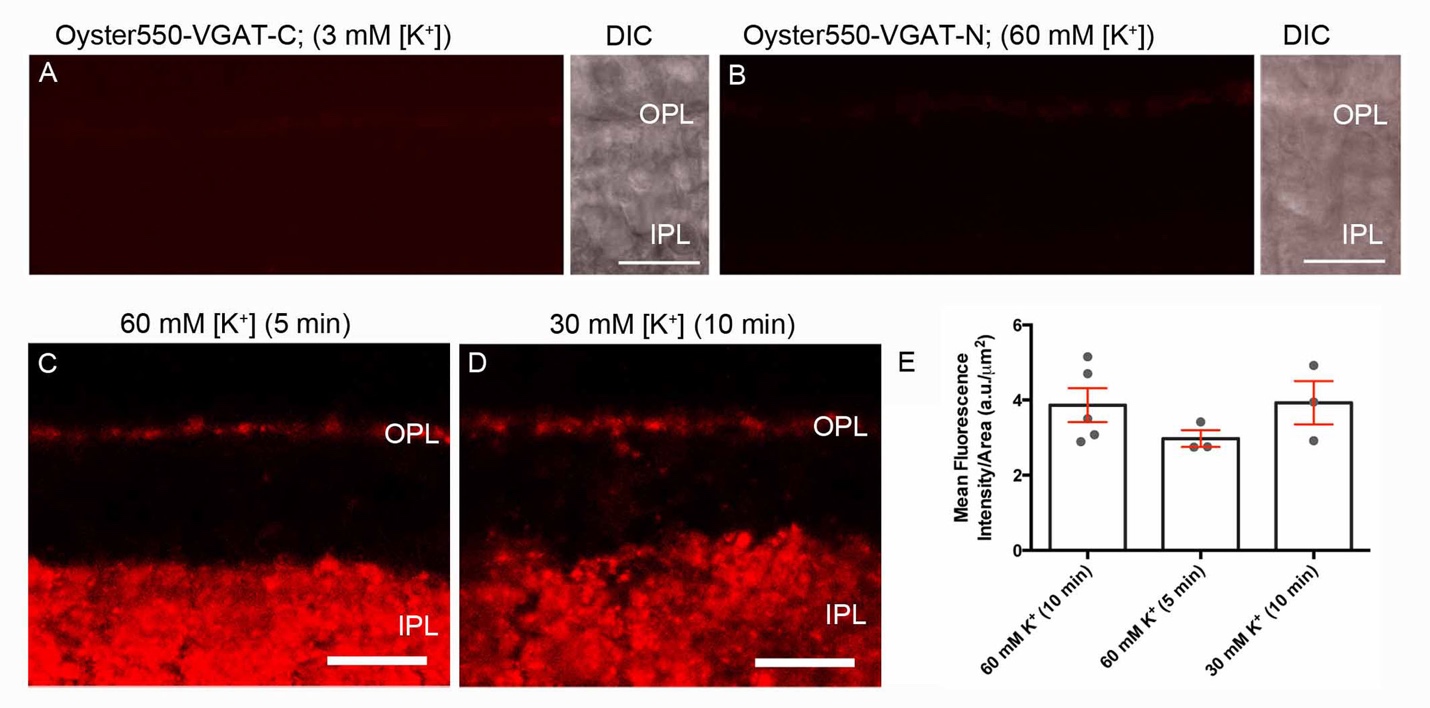


(A) Control studies showed the lack of uptake of Oyster550-VGAT-C antibody in 3 mM [K^+^]_o_.

(B) Control studies showed the lack of 60 mM [K^+^]_o_-stimulated uptake of the Oyster550-VGAT-N antibody, whose epitope is cytoplasmic and thus not exposed to the extracellular milieu.

(C) Uptake of Oyster500-VGAT-C antibody with 60 mM [K^+^]_o_ stimulation for 5 minutes.

(D) Uptake of Oyster500-VGAT-C antibody with 30 mM [K^+^]_o_ stimulation for 10 minutes.

(E) Summary of mean fluorescence intensity measured in the OPL under 60 mM [K^+^]_o_ (10 min.), 60 mM [K^+^]_o_ (5 min.) and 30 mM [K^+^]_o_ (10 min.). Measurements of mean fluorescence intensity show no significant differences in mean fluorescence intensity of slices that were incubated in 60 mM [K^+^] for 5 minutes (2.97 ± 0.22 a.u./μm^2^; N = 3; p = 0.40) or 30 mM [K^+^] for 10 minutes (3.93 ± 0.58 a.u./μm^2^; N = 3; p = 0.99) compared to the standard 60 mM [K^+^] for 10 minutes.

**Figure S2. Calcium through VGCC is necessary for depolarization-dependent Oyster550-VGAT-C antibody uptake.**


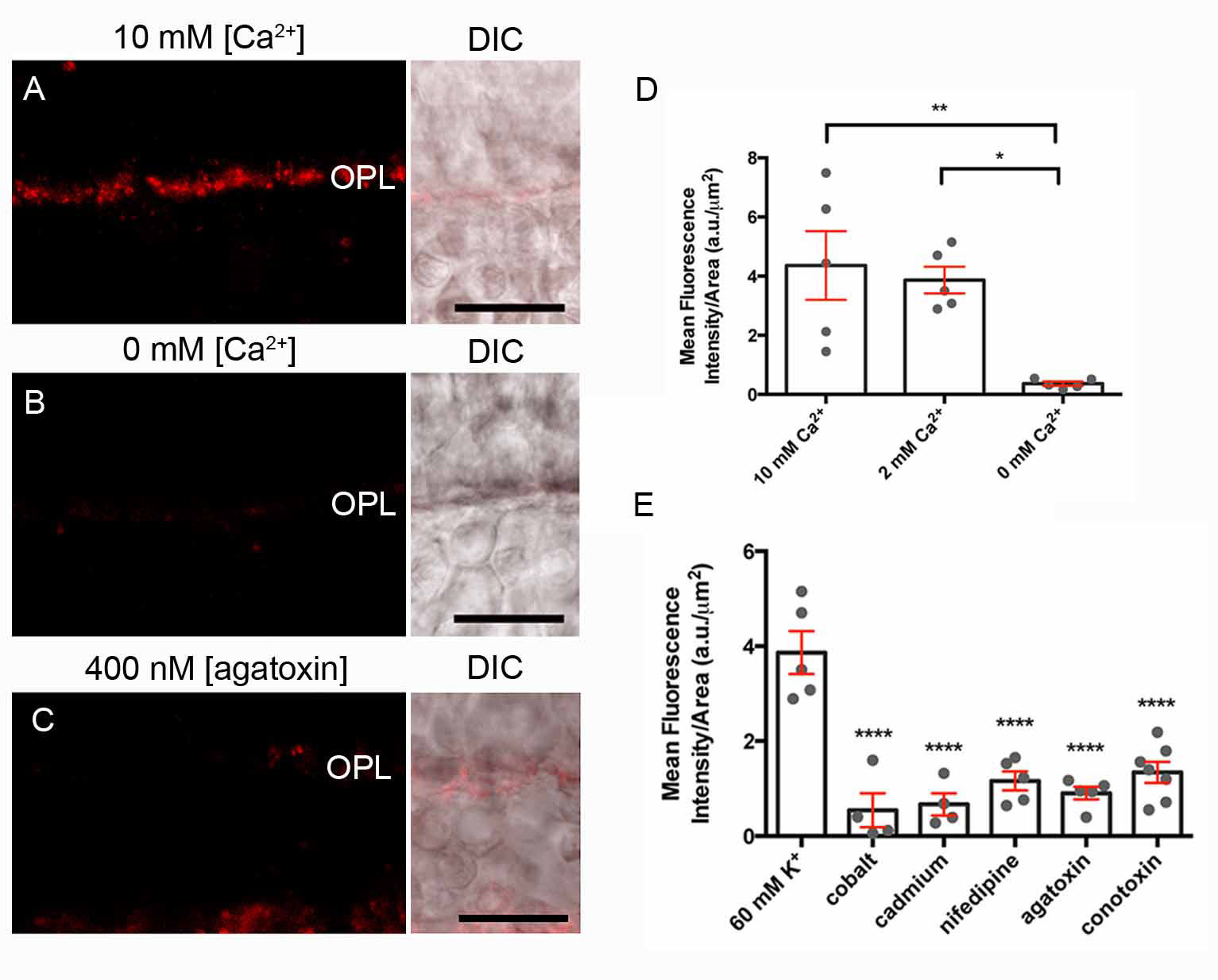


(A) Oyster550-VGAT-C antibody uptake in the OPL in 10 mM [Ca^2+^]_o_ and DIC image of the slice.

(B) Oyster550-VGAT-C antibody uptake in the OPL is minimal in nominally 0 mM [Ca^2+^]_o_ and DIC image._._

(C) Oyster550-VGAT-C antibody uptake is blocked in 400 nM ω-agatoxin, a P/Q-type VGCC antagonist and DIC image.

(D) Summary of the mean fluorescence intensity of the OPL in 10 mM [Ca^2+^]_o_, normal 2 mM [Ca^2+^]_o_, and nominally 0 [Ca^2+^]_o_. The difference in the mean fluorescence intensity levels between the 2 and 10 mM [Ca^2+^] conditions (4.36 ± 1.16 a.u./μm^2^; N = 5; p = 0.88) was not significant. In contrast, the nominally 0 mM [Ca^2+^]_o_ condition produced a significant decrease (0.36 ± 0.07 a.u./µm^2^; N = 5) compared to 2 or 10 mM [Ca^2+^] (p < 0.01* or 0.005**, respectively).

(E) General VGCC antagonists (1 mM cobalt & 200 µM cadmium) and VGCC subtype specific antagonists (10 µM nifedipine, 400 nM ω-agatoxin, & 1 µM ω-conotoxin GVIA) all diminished the VGAT-C uptake. Compared to Oyster550-VGAT-C antibody uptake in 2 mM [Ca^2+^]_o_, there is a significant reduction in fluorescence intensity with each respective Ca channel blocker (****p < 0.0001). Scale bar, 20 µm.
